# Combining ability of extra-early maturing pro-vitamin A maize (*Zea mays* L.) inbred lines and performance of derived hybrids under *Striga hermonthica* infestation and low soil nitrogen

**DOI:** 10.1371/journal.pone.0280814

**Published:** 2023-02-24

**Authors:** Solomon Akinyemi Makinde, Baffour Badu-Apraku, Omolayo Johnson Ariyo, Justina Boloebi Porbeni

**Affiliations:** 1 Department of Plant Breeding and Seed Technology, Federal University of Agriculture, Abeokuta, Ogun State, Nigeria; 2 International Institute of Tropical Agriculture, Ibadan, Oyo State, Nigeria; KGUT: Graduate University of Advanced Technology, ISLAMIC REPUBLIC OF IRAN

## Abstract

Low soil nitrogen (low-N), *Striga hermonthica* infestation and vitamin A deficiency in normal endosperm maize are major challenges confronting maize production and nutrition of the people of sub-Saharan Africa (SSA). Development of pro-vitamin A (PVA) maize hybrids with combined resistance/tolerance to the two stress factors is crucial in mitigating the food insecurity and nutrition challenges resulting from low-N deficiency and *Striga* infestation. One hundred and fifty hybrids plus six hybrid checks were evaluated under low-N, *Striga-*infested and optimal conditions in Nigeria for two years. The study examined the combining ability of the PVA inbreds in contrasting environments, classified them into heterotic groups, examined the inter-relationships of grain yield and other agronomic traits of the inbreds in hybrid combinations and assessed the performance and stability of the PVA hybrids across environments. Additive gene action conditioned the inheritance of grain yield under *Striga* infestation and optimal conditions while non-additive gene action played the major role in the inheritance of grain yield under low-N. Hybrids TZEEIOR 217 × TZEEIOR 197 and TZEEIOR 245 × TZEEIOR 195 were the top-yielding under *Striga* and low-N conditions, respectively. Inbred lines were classified into three heterotic groups. Inbreds TZEEIOR 195 and TZEEIOR 221 were identified as testers while TZEEIOR 197 × TZEEIOR 82, TZEEIOR 132 × TZEEIOR 195 and TZEEIOR 205 × TZEEIOR 221 were identified as single-cross testers. Ear aspect had direct contribution to grain yield, justifying its inclusion in the multiple trait base index used for selection of *Striga* resistant and low-N tolerant maize genotypes in SSA. Hybrids TZEEIOR 195 × TZEEIOR 149, TZEEIOR 195 × TZEEIOR 150, TZEEIOR 245 × TZEEIOR 195, TZEEIOR 30 × TZEEIOR 150 and TZEEIOR 245 × TZEEIOR 30 were high-yielding and stable across test environments. These hybrids should be tested extensively in on-farm trials and commercialized to contribute to food security in SSA.

## Introduction

Two major challenges facing countries in sub-Sahara Africa (SSA) are food insecurity and hidden hunger. Maize is an important staple crop with the potential to combat the food insecurity due to its wide acceptance and adaptability in the sub region [1]. However, maize-based diets tend to be deficient in pro-vitamin A (PVA) [2]. Consequently, over-dependence of people on maize-based diets alone results in poor health including stunted growth, reduced capacity for physical activity and in extreme cases, high incidence of anaemia, corneal blindness, compromised immunity, and infant morbidity [3]. The number of pre-school children in low-income countries affected by vitamin A deficiency (VAD) is estimated to be about 160 million [4]. Africa has 4.4% cases of maternal night blindness in the world while Nigeria accounts for 2.4% of the cases in Africa [4]. There is therefore a need for the development and commercialization of high yielding maize with elevated levels of PVA to improve nutrition in SSA.

The savannah agro-ecology of SSA has the highest potential for maize production and productivity because of its high solar radiation, low night temperatures and low pest and disease pressure [5,6]. However, production and productivity are hampered in this agro-ecology by several biotic and abiotic factors including *Striga hermonthica* (Del.) Benth. parasitism and low soil nitrogen (low -N) among others [7]. The major abiotic problem to cereal production in Africa is low soil fertility which also supports *Striga* parasitism. Long years of farming with inadequate use of fertilizer have resulted in soil nitrogen depletion in SSA [8,9]. The estimated annual loss of maize yield due to low-N stress varies from 10 to 50% [10]. Studies conducted in WCA by Badu-Apraku et al. [11] have reported 40% reduction in grain yield due to low-N and 42–65% due to *Striga* infestation. The effects of low N and *Striga* parasitism could be mitigated through the development of maize hybrids with combined tolerance to low-N and resistance to *Striga hermonthica* [12]. Therefore, development and commercialization of high yielding maize hybrids with multiple stress tolerance along with elevated levels of PVA is the focus of the International Institute of Tropical Agriculture (IITA) Maize Improvement Program (MIP), to combat food insecurity and improve nutrition in the sub-region.

Many SSA countries have adopted hybrid maize production and several high-yielding hybrids are available to seed companies in different maturity groups [12]. The IITA-MIP has over the years developed several outstanding inbreds of extra-early and grain types with tolerance/resistance to *Striga* and low soil nitrogen. The success of a commercial hybrid program depends on the availability of information on the combining ability and heterotic patterns of inbred lines. Therefore, the new inbred lines need to be extensively tested in hybrid combinations and commercialized to increase production in the sub-region. Combining ability studies of newly developed inbred lines are routinely carried out in hybrid breeding programs to provide information on the gene action controlling the traits of inbred lines [13,14]. This facilitates the selection of promising lines for hybrid development [15–17]. Even though results of several studies have been published on the gene action controlling grain yield, *Striga* resistance, low N tolerance and other traits of maize inbred lines, the reports are contradictory. For example, results of several studies have indicated that additive gene action was more important than the non-additive in the control of *Striga* resistance [18,19]. However, results of other studies have reported the non-additive gene action to be more important in the inheritance of the trait [20,21]. It has also been documented that non-additive gene action-controlled grain yield under low-N while additive gene action-controlled grain yield under high N [22,23]. Contrarily, several other authors have reported additive gene action to govern the inheritance of grain yield under low N while non-additive gene action conditioned yield under high N [24,25].

The contradicting reports on the combining abilities of the extra-early inbred lines under *Striga* infestation and low N conditions in the different studies have been attributed to the variations in the source germplasm from which the inbred lines were derived. Therefore, there is the need to assess the combining abilities of extra-early maturing PVA inbred lines extracted from diverse germplasm sources under *Striga* infestation and low-N conditions. This will facilitate the successful development of PVA hybrids with superior grain yield under the contrasting environmental conditions. Furthermore, information relating to the combining ability and heterotic patterns of extra-early maturing *Striga*-resistant/tolerant PVA inbred lines developed in the IITA-MIP is scanty. It is therefore important to determine the combining abilities of newly developed inbred lines, identify new testers and refine existing heterotic groups. The objectives of this study were therefore to (i) determine the combining ability of extra-early PVA maize inbreds under *Striga*, low-N and optimal environments (ii) classify the inbreds into heterotic groups, (iii) examine the relationship among the agronomic traits of PVA maize hybrids under *Striga*, low-N and optimal conditions (iv) investigate the PVA contents of some selected hybrids and (v) determine the grain yield and stability of the PVA maize hybrids across low-N and *Striga* endemic environments.

## Materials and methods

Thirty PVA inbred lines extracted from the extra-early maturing PVA variety 2009 TZEE-OR1 STR (Table 1) were used for this study. The development of the variety and extraction of inbred lines were carried out by the IITA-MIP in Ibadan, Nigeria [26]. Selection of the parental lines for hybridization was based on their varying responses to *Strig*a, low-N and the PVA contents. The thirty inbred lines selected for the study were crossed using the North Carolina Design II (NCDII) method proposed by Comstock and Robinson [27]. This was done by dividing the PVA inbred lines into six sets each containing five PVA inbred lines. Each inbred line served as a male in one set and as a female in another set [24], to generate a total of 150 single-cross PVA hybrids. Hence, the variation in genotypes could be partitioned into differences between males (GCA-male) and females (GCA-female) as well as their interactions (SCA).

**Table 1:** List of the extra-early maturing PVA maize inbred lines used for the North Carolina design II studies, the pedigree information, reactions to *Striga* and provitamin A contents in preliminary studies.

S/N	Designation	Pedigree	Set	Reactions to Striga *hermonthica*	Reactions to Low-N	Provitamin A (ug/g)
**1**	TZEEIOR 6	2009 TZEE-ORI STR S6 3-1/1-1/3-1/6-1/3-1/1	1	6.00	3.57	3.07
**2**	TZEEIOR 8	2009 TZEE-ORI STR S6 3-1/1-1/3-2/6-1/4-1/2	1	3.99	1.96	3.59
**3**	TZEEIOR 9	2009 TZEE-ORI STR S6 3-1/1-1/3-2/6-3/4-2/2	1	4.10	10.07	4.04
**4**	TZEEIOR 10	2009 TZEE-ORI STR S6 3-1/1-1/3-3/6-1/3-1/1	1	3.67	7.25	3.72
**5**	TZEEIOR 205	2009 TZEE-ORI STR S6 74-1/2-1/3-1/2-2/3-1/1	1	1.15	0.38	22.58
**6**	TZEEIOR 43	2009 TZEE-ORI STR S6 8-3/3-2/3-3/5-3/3-1/1	2	6.05	1.14	4.11
**7**	TZEEIOR 48	2009 TZEE-ORI STR S6 9-1/1-1/2-1/2-2/2-1/1	2	0.03	0.26	3.42
**8**	TZEEIOR 50	2009 TZEE-ORI STR S6 9-1/1-1/2-2/2-2/2-1/1	2	4.48	1.65	2.97
**9**	TZEEIOR 108	2009 TZEE-ORI STR S6 19-1/1-2/2-1/1-1/3-1/1	2	1.47	10.56	3.53
**10**	TZEEIOR 122	2009 TZEE-ORI STR S6 30-2/2-3/4-1/1-2/2-1/2	2	1.60	2.10	2.54
**11**	TZEEIOR 68	2009 TZEE-ORI STR S6 12-1/2-3/3-1/3-2/3-2/2	3	0.78	1.45	2.60
**12**	TZEEIOR 112	2009 TZEE-ORI STR S6 20-2/2-1/2-1/1-3/4-2/2	3	0.67	3.16	2.98
**13**	TZEEIOR 149	2009 TZEE-ORI STR S6 46-2/2-3/3-2/2-1/1-1/1	3	1.32	2.33	5.05
**14**	TZEEIOR 150	2009 TZEE-ORI STR S6 47-1/2-2/3-1/1-1/3-1/2	3	2.75	3.60	6.13
**15**	TZEEIOR 202	2009 TZEE-ORI STR S6 68-3/3-1/3-1/2-3/4-1/1	3	2.72	0.42	23.96
**16**	TZEEIOR 163	2009 TZEE-ORI STR S6 53-2/2-1/2-1/2-1/2-1/1	4	4.96	2.03	3.77
**17**	TZEEIOR 189	2009 TZEE-ORI STR S6 66-1/2-2/3-1/1-2/2-1/1	4	10.04	12.60	6.54
**18**	TZEEIOR 195	2009 TZEE-ORI STR S6 68-1/3-1/3-2/2-3/3-1/1	4	10.01	3.93	4.02
**19**	TZEEIOR 82	2009 TZEE-ORI STR S6 14-2/3-1/2-1/1-3/3-2/2	4	1.00	4.15	4.47
**20**	TZEEORI 30	2009 TZEE-ORI STR S6 5-1/1-2/3-1/2-2/2-1/1	4	2.97	0.40	10.19
**21**	TZEEIOR 5	2009 TZEE-ORI STR S6 2-2/2-1/2-2/2-2/2-1/1	5	0.82	1.00	3.03
**22**	TZEEIOR 245	2009 TZEE-ORI STR S6 100-1/3-3/3-1/1-1/2-1/1	5	4.30	5.18	5.59
**23**	TZEEIOR 253	2009 TZEE-ORI STR S6 100-3/3-2/3-2/2-1/4-1/1	5	8.13	4.47	5.45
**24**	TZEEIOR 132	2009 TZEE-ORI STR S6 38-1/2-2/3-1/2-2/2-2/2	5	0.55	2.27	3.44
**25**	TZEEOR 197	2009 TZEE-ORI STR S6 68-1/3-2/3-2/2-1/3-1/1	5	6.00	4.82	8.45
**26**	TZEEIOR 212	2009 TZEE-ORI STR S6 80-1/3-1/3-1/1-2/2-2/2	6	5.70	0.59	6.13
**27**	TZEEIOR 213	2009 TZEE-ORI STR S6 80-1/3-2/3-1/2-1/5-1/1	6	7.48	0.45	6.04
**28**	TZEEIOR 215	2009 TZEE-ORI STR S6 80-1/3-3/3-2/2-1/3-1/1	6	3.90	4.64	4.51
**29**	TZEEIOR 217	2009 TZEE-ORI STR S6 80-1/3-2/3-2/2-2/3-2/2	6	1.35	15.02	6.31
**30**	TZEEIOR 221	2009 TZEE-ORI STR S6 80-3/3-1/3-1/1-1/6-1/1	6	13.56	3.83	7.54

Positive value indicated resistance/tolerance to *Striga* and Low-N stresses.

### Field evaluation

The 156 F_1_ hybrids (comprising 150 PVA hybrids and 6 hybrid checks) were evaluated under *Striga*, low-N and optimal growing conditions at Abuja, Ile-Ife and Mokwa between 2018 and 2019. The experiment under *Striga* was conducted in Abuja (2018) and Mokwa (2018 and 2019) while the low-N evaluations were conducted at Mokwa in 2018 and 2019 and Ile-Ife in 2018, and the optimal experiments at Abuja and Ile-Ife in 2018 and Mokwa in 2018 and 2019. In total, there were three evaluations each under *Striga* and low-N environments and four optimal test environments. The seeds were planted in single row-plots of 3 m long with inter-row and intra-row spacings of 0.75 m and 0.40 m, respectively. A 12 × 13 simple lattice design was used in each of the evaluations.

### *Striga* environments

A total of 30 kg ha^-1^ of N, P, and K was applied as 15-15-15 NPK 30 days after planting. The reduced rate and delay in application of fertilizer were necessary to ensure good germination of *Striga* seeds and attachment of *Striga* plants to the roots of the maize plants in *Striga* infested plots. The delayed fertilizer application exposed the maize plants to the stress conditions thereby inducing production of strigolactones to stimulate the germination of *Striga* seeds [28]. Weeds other than *Striga* were manually removed by hand pulling to control weeds other than *Striga* in the field.

### Low-N environments

Depletion of N in the low-N fields was carried out through continuous planting of maize and removal of the stover at every harvest for several years before the experiments commenced. Based on the soil-tests, NPK-fertilizer was formulated using urea, single superphosphate, and muriate-of-potash. The formulated fertilizer was applied immediately after thinning at 2 WAP to bring the levels of the total available basal N to 15 kg ha^-1^. The single superphosphate (P_2_O_5_) and the muriate of potash (K_2_O) fertilizers supplied 60 kg ha^-1^ P and K. An additional 15 kg ha^–1^ of urea was top-dressed at 4 WAP to bring the total available N to 30 kg ha^–1^. The fields were kept weed-free with the application of pre- and post-emergence herbicides (atrazine and gramozone, at the rate of 5 litres ha^-1^ each) and subsequently by manual weeding.

### Optimal test environments

Under optimal conditions, N P K (15:15:15) was applied at 2 WAP to supply 60 kg ha^−1^ each of N, P and K and top-dressed with additional 30 kg N ha^−1^ at 4 WAP. Weed control in optimal environments was as described for the low-N test environments.

### Data collection

Data were collected for grain yield and other agronomic characters on per plot basis under *Striga*, low-N and optimal research conditions as shown in Table 2.

**Table 2:** Observations made on the agronomic traits and the mode of determination.

Observation	Mode of determination
Days to Anthesis (ANTH)	Number of days from sowing to when 50% of the plants in each plot had started to shed pollen [29].
Days to Silking (DYSK)	Number of days from sowing to when 50% of the plants in each plot had visible silks on the upper ear [29].
Anthesis-silking interval (ASI)	The difference between days to 50% silking and 50% anthesis (ANTH-DYSK).
Plant Height (PHT)	The distance (cm) from the base of the plant to the height of the first tassel branch. The mean of five random plants from each plot was used.
Ear Height (EHT)	The distance (cm) from the base of the plant to the node bearing the upper ear. The mean of five random plants from each plot was used.
Plant Aspect (PASP)	The overall assessment of the general architecture of plants in a plot as they appealed to sight on a scale of 1 to 9, where 1 means excellent and 9 is poor [30,31].
Ear aspect	The overall visual appeal of the ears including the size and uniformity of ears, color and texture of grains, extent of grain filling, insect, and disease damage. Ears were rated on a scale of 1–9 where, 1 = clean, uniform, large, and well-filled ears and 9 = only one or no ears or ears with undesirable features such as diseases, small ears, and ears with poorly filled grains [30,31].
Root lodging (RL)	The percentage of plants leaning more than 30° from the vertical.
Stalk lodging (SL)	The proportion or percentage of plants with broken stalk below the ear or the stalk bending more than 45° from the upright position.
Number of ears per plant (EPP)	The total number of ears with at least one fully developed grain divided by the number of harvested plants.
Number of emerged *Striga* plants	The physical counting of the number of emerged *Striga* plants within an experimental unit. This was taken at 8 and 10 WAS [31]
Host-plant damage syndrome rating	This was rated at 8 and 10 WAP, on a scale of 1–9 where 1 = no damage, indicating normal plant growth, and 9 = complete collapse or death of the maize plant [31]
Stay-green characteristic	Plants in each experimental unit were rated together at 70 DAS, on a scale of 1–9 where 1 = 0–10% dead leaf area taken upwards from the base of the plant and 9 = 90–100% dead leaf area [32].
Field weight (FWT) (kg)	The harvested ears from each plot were dehusked and weighed with weighing scale.
Grain weight (GWT) (kg)	The shelled weight of harvested ears from each plot under low-N environments.
Moisture	Ears harvested from each plot were shelled and moisture content of grains at harvest determined using “Dickey-John MiniGAC Grain Moisture tester.”
Grain Yield (kg)	Harvested ears, adjusted to 15% moisture content to compute grain yield in kilograms per hectare (kg/ha).

### Data analyses

Data on *Striga* emergence count, ear rot, stalk and root lodging were transformed as [log (counts+1)] to reduce the heterogeneity of variances. The ANOVA for the 150 hybrids generated using the NCD II pooled over sets for each research condition [15] and across the stress conditions was carried out using the version 9.4 of SAS [33]. The genotypic component of the source of variation was partitioned into the variation due to males (sets), females (sets), and female × male (sets) interaction. The F-tests for male (sets), female (sets) and male × female (sets) mean squares were performed using male (sets) × environment, female (sets) × environment and male × female (sets) × environment mean squares, respectively. The mean squares attributable to environment × female × male (sets) were tested using the pooled error mean squares.

The following general linear model was used for the NCD II mating design:

$$X_{ijkl}=\mu +m_{i}+f_{i}+{\left(mf\right)}_{ij}+p_{ijk}+I_{l}+\varepsilon_{ijkl}$$

where X_ijkl_ = the observed value of the progeny of the i^th^ male crossed with j^th^ female in the k^th^ replication; μ = the overall population mean; m_i_ = effect of the i^th^ female; f_j_ = the effect of the j^th^ male mated to the i^th^ female; (mf)_ij_ = the interaction effect between the i^th^female and the j^th^ male; p_ijk_ = the effect of the k^th^ progeny from the cross between i^th^ female and j^th^ male; r_l_ = the effect of the l^th^ replication; ε_ijkl_ = the experimental error. The general combining ability (GCA) effects for male and female within sets (GCA_m_ and GCA_f_) and specific combining ability (SCA) for each trait were estimated according to Kearsey and Pooni [34] as shown below:

$$GCA_{m}=X_{m}-\mu$$

$$GCA_{f}=X_{f}-\mu$$

where, GCA_m_ and GCA_f_ = General combining ability effects of male and female parents respectively; X_m_ and X_f_ = Average performance of a line when used as a male and female in crosses, respectively and μ = Overall mean of crosses in the set.

Standard errors (SE) for testing significance of GCA_m_ and GCA_f_ estimates, for traits of genotype, were computed from the mean squares of GCA_m_ × environment and GCA_f_ × environment, respectively as follows:

$$SE\;for\;GCA_{m}=\surd MSm\times e/(f\times e\times r)$$

$$SE\;for\;GCA_{f}=\surd MSf\times e/(m\times e\times r)$$

where, MSm × e and MSf × e were the mean squares of the interaction between male and environment as well as female × environment, respectively; f, m, r, and e were the number of females, males, replicates, and environments, respectively.

A multiple trait base index (MI) that integrated grain yield with the number of emerged *Striga* plants, *Striga* damage rating, plant and ear aspects, delayed leaf senescence, anthesis-silking interval and number of ears per plant was used to select the best performing hybrids across optimal, *Striga* and low-N conditions [5]. The means, adjusted for block effects of each genotype for each measured variable was standardized to minimize the effects of the different scales. A positive multiple trait base index value therefore indicated tolerance/resistance of the genotype to both *Striga* and low-N, while negative values indicated susceptibility to the stresses. The multiple trait base index was computed as follows:

MI = (2 × YLD) + EPP–EASP–PASP—STGR–RAT1 –RAT2 –(0.5 × C01)–(0.5 × C02)

On the other hand, the base indices for *Striga* and Low-N were computed as STRBI = 2.0 YLD + 1.0 EPP–(RAT1 + RAT2)– 0.5 (C01 + C02) and LNBI = 2.0 YLD + EPP–STGR–ASI—PASP–EASP, respectively to select superior hybrids under the respective stress conditions.

Where: MI = Multiple trait base index

STRBI = Base index for *Striga*

LNBI = Base index for Low-N

YLD = grain yield across research conditions

EPP = number of ears per plant across research conditions

EASP = Ear aspect across research conditions

PASP = Plant aspect across low-N and optimal conditions

STGR = Stay green characteristic across low-N conditions

RAT1 and RAT2 = *Striga* damage rating at 8 and 10 WAP across *Striga* infested conditions

C01 and C02 = Number of emerged *Striga* plants at 8 and 10 WAP across *Striga* -infested conditions.

### Heterotic grouping of inbred lines

The heterotic grouping method based on general combining ability of multiple traits (HGCAMT) proposed by Badu-Apraku et al. [7,13] has been an effective method for classifying inbreds into heterotic groups [26]. The HGCAMT method was therefore used to assign the 30 extra-early maturing PVA lines into heterotic groups. The grouping was done by standardizing the GCA effects with a mean of zero and standard deviation of 1 for measured traits with significant genotypic mean squares and GCA across research conditions using the following statistical model:

$$Y=\sum_{i=1}^{n}(\frac{\gamma_{i}-{ȳ}_{i}}{s})+\varepsilon_{{ij}^{}}$$

where Y is HGCAMT, which is the genetic value measuring relationship among genotypes based on the GCA of multiple traits i to n; *γ_i_* is the individual GCA effects of genotypes for trait i, ȳ_*i*_ is the mean of GCA effects across genotypes for trait i; s is the standard deviation of the GCA effects of trait i, and *ε_ij_* is the residual of the model associated with the combination of inbred i and j. The standardized GCA effects were converted to Euclidean distance using PROC DISTANCE procedure in SAS and subsequently subjected to Ward’s minimum variance cluster analysis using PROC CLUSTER procedure in SAS [33].

The associations among traits under *Striga*, low-N, combined *Striga* and low-N and optimal conditions were measured using the stepwise multiple regression and sequential path diagrams using the method proposed by Mohammadi et al. [35]. The stepwise multiple regression analysis was carried out using the Statistical Package for Social Sciences, SPSS version 17.0 [36] to identify the first-, second-, and third-order predictor traits based on their contributions to the total variation in grain yield with minimized multicolinearity [37–39]. Firstly, all other traits were regressed on grain yield to identify those with significant contributions to grain yield at P ≤ 0.05 as first-order traits. The rest of the traits were regressed on each of the first order traits and those with significant contributions to grain yield through the first-order traits were classified as second-order traits. The procedure was repeated, and the remaining traits categorized into subsequent orders. The standardized b values generated by the stepwise regression analysis were the path co-efficients [35,38,39]. The significance of the path coefficients was determined in the stepwise multiple regression analysis using t-test at 5% probability level and traits with significant path co-efficients were retained. Also, the relationships among traits within an order of traits were determined using the Spearman correlation analysis implemented in SAS version 9.4 [33].

### Production of kernel samples for carotenoid analyses

Thirty-two selected hybrids and 3 hybrid checks were planted under well-watered growing conditions in July 2019 at the Mokwa research station of IITA. The hybrids were planted in single row plots, each 1 m long with inter-row and intra-row spacings of 0.75 and 0.20 m, respectively. Two seeds were planted per hill and thinned to one plant per hill at 2 WAP to obtain five plants per row. Seed samples were produced by controlled self-pollination of the plants in each plot. Ears of self-pollinated F_1_ hybrids in each plot were harvested, labelled, and dried under ambient temperature with minimal exposure to direct sunlight and shelled separately. The grains were stored in the IITA cold store (-10° C) for six months after which 100 kernels were sampled per hybrid and sent to the IITA nutritional laboratory in Ibadan for carotenoid analyses.

### Carotenoid analysis using high performance liquid chromatography (HPLC)

Carotenoids were extracted and quantified using HPLC at the IITA Nutritional Laboratory, Ibadan, Nigeria. The protocol described by Howe and Tanumihardjo [39] was used for the extraction and analysis of the carotenoids. Finely ground 0.6 g sample of each entry was transferred into a 50 ml glass centrifuge tube to which 6 ml of ethanol plus 0.1% butylated hydroxyl toluene had been added. After vortexing for 15 seconds, the tubes were placed in 85°C water bath for 5 minutes and 500 μl of 80% potassium hydroxide (w:v) was added. The samples were vortexed for 15 seconds and put back in the water bath for 10 minutes with vortexing at approximately 5 minutes interval. They were then immediately placed on ice and 3 ml ice cold deionized water was added, vortexed for 15 seconds, followed by addition of 200 μl of the internal standard β-Apo-8’-carotenal and 4 ml hexane. After vortexing and centrifugation, the top hexane layer formed was transferred into a new test tube. The hexane extraction was repeated twice, adding 3 ml hexane each time. Samples were allowed to dry down completely under nitrogen gas using a Turbovap LV concentrator and reconstituted in 500 μl of 50:50 Methanol: Dichloroethane. Following vortexing and centrifuging, the extracts were transferred to HPLC vials placed in the autosampler tray and 50 μl aliquots of each extract were injected into an HPLC system. The Waters HPLC system was operated with Empower 1 software which included a 717 Plus auto sampler with temperature control set at 5°C, a Waters 1525 binary HPLC pump, and a 2996 photodiode array detector for carotenoid quantification. Carotenoids were separated on a 3 μm C30 YMC Carotenoid Column (4.6 × 250 mm) eluted with a mobile phase of methanol/water (92:8 v/v) with 10 mM ammonium acetate as solvent A, and 100% methyl tertiary butyl ether as solvent B.

The gradient was applied for 30 minutes from 70% solvent A: 30% solvent B, to 40% solvent A: 60% solvent B. The flow rate was 1.0 mL/min. To maximize detection of carotenoids, the absorbance was measured at 450 nm. Beta-carotene (cis and trans isomers), α-carotene, β-cryptoxanthin, zeaxanthin, and lutein were assayed based on calibrations using the respective external standards. Total carotenoids were computed as the sum of concentrations of α-carotene, β-carotene, lutein, Zeaxanthin and β-cryptoxanthin. PVA was computed as the sum of β-carotene, and half of each of β-cryptoxanthin and α-carotene contents, since β-cryptoxanthin and α-carotene contribute half (50%) of the value of β-carotene as PVA [40]. Values of all carotenoids for each sample were obtained from two independent measurements for statistical analysis.

## Results

### Analysis of variance of grain yield and other agronomic traits of extra-early maturing PVA hybrids under *striga*, low-N and optimal conditions

Under *Striga* environments, the analysis of variance showed significant (P < 0.05 or P < 0.01) differences in the mean squares for Env, Hybrid, GCA-male and GCA-female for grain yield and other measured traits (Table 3). The SCA mean squares were significant for measured traits except the mean squares for grain yield, anthesis-silking interval (ASI), *Striga* damage at 10WAP (RAT2) and number of ears per plant (EPP). Similarly, Hybrid × Env was significant for grain yield and most measured traits except EPP. In addition, the grain yield and most measured traits had significant mean squares for Env × GCA-male, Env × GCA-female and Env × SCA with exceptions of *Striga* count at 8WAP (C01) and EPP for Env × GCA-male, EPP and Ear aspect (EASP) for Env × GCA-female and ASI, RAT2, EPP and EASP for Env × SCA.

**Table 3:** Mean squares of grain yield and other agronomic traits of extra-early maturing PVA maize hybrids evaluated under *Striga* conditions.

Source	Degree of freedom	Grain yield (kg ha^-1^)	ASI	RAT1	RAT2	C01	C02	EPP	EASP (1–9)
**ENVIRONMENT(ENV)**	2	540384528**	957.79**	5.47**	9.17**	5.54**	19.67**	2.52**	5.95**
**SET**	5	15805774**	7.13	9.14**	11.53**	0.78**	0.41**	0.40**	9.62**
**ENV × SET**	10	1133414	4.83	1.00**	1.07*	0.08	0.05	0.11	0.52
**REP(ENV × SET)**	15	488354	3.31	0.46	0.56	0.06	0.04	0.06	0.75**
**BLOCK(ENV × REP)**	72	2415215**	3.61	1.23**	1.39**	0.08*	0.06**	0.16**	1.56**
**HYBRID**	155	2765767**	6.66**	1.48**	1.92**	0.19**	0.12**	0.14**	1.52**
**GCA-male**	24	3719661**	8.88**	2.10**	3.14**	0.34**	0.19**	0.15*	1.71**
**GCA-female**	24	5618422**	12.82**	2.61**	3.47**	0.27**	0.19**	0.24**	3.04**
**SCA**	96	987666	4.39	0.60**	0.58	0.09**	0.07**	0.10	0.62**
**HYBRID × ENV**	310	1108962**	5.77**	0.63**	0.65**	0.07**	0.07**	0.09	0.55**
**ENV × GCA-male**	48	1212558*	6.41*	0.70**	0.75*	0.07	0.12**	0.10	0.66*
**ENV × GCA-female**	48	1584275**	9.79**	0.67**	0.68*	0.08*	0.08**	0.10	0.56
**ENV × SCA**	192	1040364**	4.54	0.54*	0.58	0.08**	0.06**	0.09	0.51
**Error**	360	834501	4.24	0.41	0.48	0.05	0.03	0.09	0.42

ASI = Anthesis-silking interval, RAT1 and RAT 2 = *Striga* damage rating at 8 and 10 WAP, C01 and C02 = Emerged *Striga* plants at 8 and 10 WAP, EPP = number of ears per plant, EASP = Ear aspect, GCA-male = general combining ability effects of male, GCA-female = general combining ability effects of female, SCA = specific combining ability effect

*, ** = Significant at 0.05 and 0.01 probability levels, respectively.

Under low-N conditions, the results revealed that Env, Sets and Hybrids for grain yield and most other measured traits were significant except for ASI for Sets and Hybrids (Table 4). Similarly, GCA-male and GCA-female were significant for grain yield and most other measured traits under low-N environments, with the exception of GCA-male for ASI and stay-green characteristic (STGR) and GCA-female for ASI. The SCA showed significant differences for grain yield. However, Hybrid × Env mean squares showed significant variation for grain yield and other measured traits. Furthermore, there was significant variation for Hybrid × Env, Env × GCA-male, Env × GCA-female and Env × SCA for grain yield and other measured traits except the STGR for Env × GCA-male, ear aspect (EASP) for Env × GCA-female and EPP, STGR and EASP for SCA x Env effects.

**Table 4:** Mean squares of grain yield and other agronomic traits of extra-early maturing PVA maize hybrids evaluated under low-N environments in 2018 (Ile-Ife and Mokwa) and 2019 (Mokwa).

Source	Degree of freedom	Grain yield (kg ha^-1^)	ASI	EPP	STGR (1–9)	EASP (1–9)
**ENVIRONMENT (ENV)**	2	233526260**	225.70**	0.85**	449.84**	9.64**
**SET**	5	3626492**	0.69	0.12**	1.24*	5.14**
**ENV × SET**	10	1566715**	1.35	0.03	1.52**	0.90
**REP (ENV × SET)**	15	569587*	0.36	0.01	0.80	0.80
**BLOCK (ENV × REP)**	72	987795**	1.40	0.05*	1.19**	0.89
**HYBRID**	155	1051646**	1.85	0.05**	0.85**	0.97**
**GCA-male**	24	1028799**	2.05	0.07**	0.68	0.84*
**GCA-female**	24	1574365**	1.48	0.09**	2.15**	1.70**
**SCA**	96	774741**	1.30	0.03	0.51	0.58
**HYBRID × ENV**	310	773557**	2.02**	0.04**	0.78**	0.61*
**ENV × GCA-male**	48	927835**	2.08**	0.06**	0.64	0.71*
**ENV × GCA-female**	48	1037285**	1.92**	0.05**	1.82**	0.55
**ENV × SCA**	192	605133**	1.22**	0.03	0.48	0.57
**Error**	360	281354	1.14	0.03	0.54	0.49

ASI = Anthesis-silking interval, EPP = number of ears per plant, STGR = Stay green characteristic, EASP = Ear aspect, GCA-male = general combining ability of male, GCA-female = general combining ability of female, SCA = specific combining ability

*, ** = Significant at 0.05 and 0.01 probability levels, respectively.

In the four optimal conditions, results revealed significant (P ≤ 0.01 or P ≤ 0.05) variation for env, set, env × set, hybrid, and hybrid × env for grain yield and other measured traits except ASI for set and hybrid and EPP for hybrid x env (Table 5). The GCA-male and GCA-female showed significant differences for grain yield and most other measured traits while SCA was signinificant for grain yield. Env × GCA-male, Env × GCA-female, and Env × SCA were also significant for grain yield and other measured traits except EPP and EASP for Env × GCA-male. Other exceptions were ASI, and EPP for Env × GCA-female as well as EPP and EASP for Env × SCA.

**Table 5:** Mean squares of grain yield and other agronomic traits of extra-early maturing pro-vitamin A maize hybrids evaluated under optimal environments at Mokwa (2018 and 2019), Ile-Ife and Abuja (2018).

Source	DF	Grain Yield (kgha^-1^)	ASI	EPP	EASP
**Environment (ENV)**	3	232777213**	112.18**	0.44**	53.68**
**SET**	5	11909315**	0.63	0.05*	6.11**
**ENV × SET**	15	4784832**	1.72	0.02	1.89**
**REP (ENV× SET)**	20	1035878	1.39	0.02	0.76
**BLOCK (ENV × REP)**	96	1670675**	1.51*	0.03	1.45**
**HYBRID**	155	4790257**	1.18	0.03**	1.58**
**GCA-male**	24	4959132**	1.71*	0.03	1.89**
**GCA-female**	24	8337040**	0.64	0.04*	3.43**
**SCA**	96	3142637**	1.21	0.02	0.8
**HYBRID × ENV**	465	2293033**	1.36**	0.02	0.84*
**ENV × GCA-male**	72	2210249**	1.84**	0.02	0.87
**ENV × GCA-female**	72	2495348**	1.14	0.02	0.96*
**ENV × SCA**	288	1931990**	1.32*	0.02	0.68
**Error**	480	730660**	1.09	0.02	0.68

ASI = Anthesis-silking interval, EPP = Number of ears per plant, EASP = Ear aspect

* and ** = significant at 0.05 and 0.01 probability levels, respectively.

### Proportionate contributions of GCA and SCA sums of squares under *striga*, low-N and optimal conditions

Under artificial *Striga* infestation, the gross contributions of GCA (GCA-male + GCA-female) to hybrid variation ranged from 49.9% (EPP) to 73.95% (RAT2) whereas the SCA sum of squares varied from 26.05% for RAT2 to 50.10 for EPP (Table 6). The GCA sum of squares accounted for about 59 to 74% of the total variation for number of emerged *Striga* plants at 10 WAP (C02), number of emerged *Striga* plants at 8 WAP (C01), *Striga* damage at 8 WAP (RAT1), grain yield and RAT2. In most cases, the contribution of GCA-female sum of squares was higher than GCA-male and SCA sum of squares for grain yield and most other mesured traits whereas, the contributions of SCA sum of squares for EPP was higher than the percentage contribution of the GCA sum of squares.

**Table 6:** Proportion of total-genotypic sums of squares for grain-yield and other agronomic traits of extra-early maturing provitamin A maize-inbred lines attributable to general combining ability (GCA-male and GCA-female) and specific combining-ability (SCA) effects of the inbred lines and hybrids, respectively under *Striga*, low-N and optimal conditions in Nigeria, 2018–2019.

Traits	*Striga*			Low-N			Optimal		
	GCAm	GCAf	SCA	GCAm	GCAf	SCA	GCAm	GCAf	SCA
Grain yield (kg ha^-1^)	27.99	42.28	29.73	18.04	27.61	54.35	19.17	32.23	48.60
Anthesis-silking interval (ASI)	22.62	32.65	44.72	23.44	16.97	59.59	23.83	8.88	67.29
Ear aspect (EASP) (1–9)	23.60	42.02	34.38	17.26	35.06	47.69	22.24	40.34	37.42
Ears per plant (EPP)	19.32	30.58	50.10	25.85	31.01	43.14	17.53	24.26	58.21
Stay green characteristic (STGR)	-	-	-	14.10	44.20	41.70	-	-	-
*Striga* damage rating at 8 WAP (RAT1)	29.61	36.72	33.67	-	-	-	-	-	-
*Striga* damage rating at 10 WAP (RAT2)	35.15	38.80	26.05	-	-	-	-	-	-
Emerged *Striga* plants at 8 WAP (C01)	34.33	27.91	37.76	-	-	-	-	-	-
Emerged *Striga* plants at 10 WAP (C02)	29.18	29.75	41.07	-	-	-	-	-	-

GCAm = General combining ability of male, GCAf = general combining ability of female, SCA = Specific combining ability

*, ** = Significant at 0.05 and 0.01 probability levels, respectively.

Under low-N conditions, the contributions of GCA sum of squares ranged from 40.41% for ASI to 58.30% for stay green characteristic (STGR) while that of SCA sum of squares varied from 41.70% (STGR) to 59.59% (ASI). Higher contribution of GCA (GCA-male + GCA-female) sum of squares were recorded for EASP, EPP and STGR than their corresponding SCA sum of squares. In contrast, the contribution of SCA sum of squares were higher than the corresponding GCA sum of squares for ASI and grain yield.

The contributions of GCA sum of squares to the overall genotypic variation among hybrids under optimal conditions varied from 32.71% to 62.58% for ASI and EASP, respectively while SCA sum of squares varied from 37.42% for EASP to 67.29% for ASI. Higher contribution of GCA (GCA-male + GCA-female) were recorded for grain yield and EASP while the contribution of SCA sum of squares were higher for ASI and EPP than their corresponding GCA.

### General combining ability effects of the extra-early provitamin A inbred lines

Under *Striga-*infested environments, the GCA-male effects for grain yield varied from -781.94 for TZEEIOR 189 to 727.51 for TZEEIOR 221 (Table 7). Parental lines TZEEIOR 205, TZEEIOR 150, TZEEIOR 82 and TZEEIOR 221 had significant and positive GCA-male effects for grain yield. However, significant, and negative GCA-male effects were observed for RAT1 for inbreds TZEEIOR 205, TZEEIOR 108 and TZEEIOR 5. Inbreds TZEEIOR 205, TZEEIOR 150, TZEEIOR 202, TZEEIOR 195, TZEEIOR 82 and TZEEIOR 221 had significant and negative GCA-male effects for RAT2. Similarly, significant, and negative GCA-male effects were recorded for inbreds TZEEIOR 205, TZEEIOR 48, TZEEIOR 202, TZEEIOR 195, TZEEIOR 253 and TZEEIOR 212 for C01 while inbreds TZEEIOR 6 and TZEEIOR 253 recorded negative and significant GCA-male effects for C02. Contrarily, the GCA-female effects for grain yield ranged from -911.68 for TZEEIOR 82 to 730.82 for TZEEIOR 195, with TZEEIOR 9, TZEEIOR 108, TZEEIOR 195 and TZEEIOR 253 recording positive and significant GCA-female effects. Five inbreds (TZEEIOR 43, TZEEIOR 189, TZEEIOR 195, TZEEIOR 253 and TZEEIOR 221) showed significant and negative GCA-female effects for RAT1, while negative and significant GCA-female for RAT2 were observed for TZEEIOR 108, TZEEIOR 149, TZEEIOR 150, TZEEIOR 253 and TZEEIOR 221. Parental lines TZEEIOR 8, TZEEIOR 43, TZEEIOR 149, TZEEIOR 150, TZEEIOR 195 and TZEEIOR 221 displayed negative and significant GCA-female effects for C01. Additionally, five inbreds (TZEEIOR 8, TZEEIOR 149, TZEEIOR 150, TZEEIOR 195 and TZEEIOR 221) had negative and significant GCA-female effects for C02.

**Table 7:** General combining ability effects of grain yield and other agronomic traits of PVA maize inbred lines evaluated under *Striga*, low-N and optimal conditions.

Inbreds	†Striga		†Low-N		†Optimal		STGR		RAT1		RAT2		C01		C02	
	GCA-male	GCA-female	GCA-male	GCA-female	GCA-male	GCA-female	GCA-male	GCA-female	GCA-male	GCA-female	GCA-male	GCA-female	GCA-male	GCA-female	GCA-male	GCA-female
**TZEEIOR 6**	-366.15*	165.84	-111.55	-15.78	7.16	204.11	-0.02	-0.47*	0.28*	0.01	0.42**	0.17	-0.04	0.05	-0.14*	0.03
**TZEEIOR 8**	47.39	-493.09*	140.54	176.99	-554.97*	-694.93**	0.07	-0.29	0.23	0.25	0.09	0.21	0.09*	-0.12*	0.06	-0.10*
**TZEEIOR 9**	-39.8	465.40*	-113.64	-364.92*	96.62	-288.36	-0.17	0.45	-0.11	-0.05	-0.18	-0.13	0.09*	-0.02	0.01	-0.04
**TZEEIOR 10**	-67.01	-20.44	-322.25	-25.89	-33.10	38.02	-0.03	0.01	0.04	-0.05	0.13	-0.09	0.02	0.03	0.06	0.02
**TZEEIOR 205**	425.57*	-117.70	406.89*	229.59	484.29*	741.16**	0.15	0.30	-0.45**	-0.16	-0.47**	-0.16	-0.16**	0.06	0.01	0.08
**TZEEIOR 43**	-254.67	256.65	-47.52	370.20	-320.03	489.28*	0.83**	-0.25	0.15	-0.44**	0.33*	-0.18	0.08*	-0.10*	0.03	-0.06
**TZEEIOR 48**	97.87	-23.25	-201.89	344.84*	50.76	286.84	0.84**	-0.36	0.24	0.24	0.03	0.14	-0.10*	0.11*	-0.09	0.05
**TZEEIOR 50**	-120.95	5.60	114.53	-343.24*	-293.04	230.24	1.15**	0.00	-0.03	0.08	0.09	0.00	-0.06	0.08	-0.06	0.05
**TZEEIOR 108**	185.69	663.65**	44.74	311.08	-273.17	340.55	1.14**	0.20	-0.33*	-0.40	-0.30	-0.56**	0.04	-0.06	0.05	-0.01
**TZEEIOR 122**	92.06	-902.65**	90.14	-682.88**	835.48**	-1346.90**	1.05**	0.42	-0.02	0.52**	-0.15	0.61**	0.04	-0.03	0.07	-0.03
**Inbreds**	**†Striga**		**†Low-N**		**†Optimal**		**STGR**		**RAT1**		**RAT2**		**C01**		**C02**	
	**GCA-male**	**GCA-female**	**GCA-male**	**GCA-female**	**GCA-male**	**GCA-female**	**GCA-male**	**GCA-female**	**GCA-male**	**GCA-female**	**GCA-male**	**GCA-female**	**GCA-male**	**GCA-female**	**GCA-male**	**GCA-female**
**TZEEIOR 68**	-119.41	151.49	62.82	30.90	49.72	-169.49	-0.04	-0.38	0.08	-0.22	0.22	-0.20	-0.03	0.15**	0.07	0.15**
**TZEEIOR 112**	-211.86	-762.72**	178.55	84.49	-28.51	-588.30*	0.11	0.34	0.12	0.29*	0.31*	0.68**	0.03	0.06	0.04	0.05
**TZEEIOR 149**	-125.06	341.88	124.56	26.66	-75.34	200.16	0.01	0.21	0.28*	0.04	0.29*	-0.32*	0.10	-0.11*	0.01	-0.11*
**TZEEIOR 150**	359.98*	292.60	-245.70	-6.19	-88.30	63.37	-0.16	0.02	-0.25	-0.16	-0.35*	-0.29*	0.03	-0.13*	-0.01	-0.10*
**TZEEIOR 202**	96.35	-23.26	-120.22	-135.86	142.43	494.25*	0.09	-0.19	-0.23	0.04	-0.47**	0.13	-0.13**	0.03	-0.10	0.00
**TZEEIOR 163**	-56.45	-332.86	-306.08	-138.91	-165.74	-222.98	-0.15	0.23	-0.15	-0.11	-0.10	0.05	0.07	0.01	0.06	-0.07
**TZEEIOR 189**	-781.94**	518.93*	115.16	36.10	-648.09**	-128.16	0.52**	0.27	0.63**	-0.49**	0.86**	-0.17	0.20**	0.10	0.06	0.13*
**TZEEIOR 195**	330.25	730.82**	105.17	-111.62	526.89*	-222.23	-0.05	-0.05	-0.17	-0.61**	-0.33*	-0.60**	-0.14**	-0.18**	-0.08	-0.10*
**TZEEIOR 82**	492.13*	-911.68**	10.92	82.84	466.00*	888.74**	-0.16	-0.20	-0.09	0.77**	-0.37*	0.83**	-0.07	0.12*	-0.04	0.09
**TZEEIOR 30**	16.01	-5.21	74.83	131.60	-179.06	-315.38	-0.15	-0.26	-0.22	0.01	-0.06	-0.10	-0.05	-0.05	0.00	-0.06
**Inbreds**	**†Striga**		**†Low-N**		**†Optimal**		**STGR**		**RAT1**		**RAT2**		**C01**		**C02**	
	**GCA-male**	**GCA-female**	**GCA-male**	**GCA-female**	**GCA-male**	**GCA-female**	**GCA-male**	**GCA-female**	**GCA-male**	**GCA-female**	**GCA-male**	**GCA-female**	**GCA-male**	**GCA-female**	**GCA-male**	**GCA-female**
**TZEEIOR 5**	118.13	-31.81	-115.59	-271.74	-54.68	-682.83**	0.15	0.2	-0.35*	-0.06	-0.27	-0.02	0.01	-0.06	-0.1	-0.05
**TZEEIOR 245**	296.89	-109.81	-37.29	162.1	375.52	76.42	0.07	-0.35	0.19	-0.01	-0.03	-0.18	0.08*	0.13**	0.14*	0.08
**TZEEIOR 253**	-11.87	486.75**	95.66	-54.43	-385.92	163.65	-0.05	0.06	-0.22	-0.36*	-0.18	-0.29*	-0.21**	-0.03	-0.15*	-0.01
**TZEEIOR 132**	-760.2**	-369.75	72.83	-80.63	282.74	497.75*	-0.13	0.24	0.40*	0.36*	0.53**	0.40**	0.16**	-0.01	0.13*	-0.02
**TZEEIOR 197**	357.05	24.62	-15.6	244.71	-217.67	-55	-0.04	-0.15	-0.01	0.06	-0.05	0.09	-0.05	-0.03	-0.01	0.01
**TZEEIOR 212**	188.99	-391.3	-187.02	-2.11	-456.11*	-75.36	0.22*	-0.25	-0.02	0.09	0.01	0.21	-0.22**	-0.04	-0.1	0.01
**TZEEIOR 213**	-189.9	-151.4	-208.12	-54.47	188.53	-208.72	-0.01	0.15	-0.19	0.25	-0.03	0.24	0.15**	0.13*	0.12*	0.03
**TZEEIOR 215**	-40.79	-20.38	123.28	149.44	23.9	-78.07	0.03	-0.05	-0.01	0.02	-0.01	-0.11	0.08*	0.06	0.01	0.12*
**TZEEIOR 217**	-685.8**	211.02	-127.72	-10.73	-293.21	59.1	-0.09	-0.08	0.43**	-0.04	0.35*	-0.01	-0.01	0.05	0.03	0.02
**TZEEIOR 221**	727.51**	352.06	399.58*	-82.12	536.89*	303.04	-0.14	0.23	-0.22	-0.32*	-0.33*	-0.33*	0.01	-0.20**	-0.06	-0.18**
**S.E**	179.82	205.54	157.3	166.32	210.25	223.4	0.13	0.22	0.14	0.13	0.14	0.13	0.04	0.05	0.06	0.05

*^,^ ** = Significant at 0.05 and 0.01 probability levels, respectively

**†,** STGR, RAT1, RAT2, C01 and C02 = Grain yield, Stay green characteristic, *Striga* damage rating and *Striga* emergence count at 8 and 10 weeks after planting, respectively; EASP = ear aspect.

The GCA-male effects for grain yield under low-N environments varied from -322.50 for TZEEIOR 10 to 406.89 for TZEEIOR 205. Inbreds TZEEIOR 205 and TZEEIOR 221 had significant and positive GCA-male effects for grain yield under low N environments. In contrast, the GCA-female effects for grain yield under low-N ranged from -682.88 for inbred TZEEIOR 122 to 370.20 for TZEEIOR 43. Inbred TZEEIOR 48 had positive and significant GCA-female effects for grain yield while TZEEIOR 6 had negative and significant GCA-female effects for STGR.

### Performance of extra-early maturing PVA hybrids under *striga*-infested, low-N and optimum conditions

Under *Striga* infested conditions, grain yield of PVA maize hybrids varied from 1629 kg ha^-1^ for TZEEIOR 82 × TZEEIOR 68 to 5162 kg ha^-1^ for TZEEIOR 217 × TZEEIOR 197 with a mean of 3053 kg ha^-1^ (Table 8). Under low-N, grain yield ranged from 955 kg ha^-1^ for TZEEIOR 122 × TZEEIOR 8 to 3527 kg ha^-1^ for TZEEIOR 245 × TZEEIOR 195 with a mean of 2415 kg ha^-1^. The mean grain yield under optimal condition was 5481 kg ha^-1^ with hybrids TZdEEI 7 × TZdEEI 12 (3192 kg ha^-1^) and TZEEIOR 82 × TZEEIOR 68 (6933 kg ha^-1^) recording the least and highest grain yield, respectively. The highest yielding hybrids outyielded the best hybrid checks under *Striga*, low-N and optimal conditions by 24, 29 and 2%, respectively.

**Table 8:** Grain yield of selected PVA maize hybrids across *Striga*, low-N and optimal conditions.

	Grain yield (kg ha^-1^)			Grain yield reduction (%)				
**HYBRID**	*Striga*	Low-N	Optimal	*Striga*	Low-N	STRBI	LNBI	MI
**TZEEIOR 48 x TZEEIOR 9**	2335	2340	5789	59.7	59.6	-9.2	-3.0	-8.9
**TZEEIOR 122 x TZEEIOR 6**	1987	1661	3566	44.3	53.4	-10.9	-12.3	-22.4
**TZEEIOR 122 x TZEEIOR 8**	2180	955	3483	37.4	72.6	-8.2	-17.8	-21.0
**TZEEIOR 122 x TZEEIOR 9**	1801	1318	3276	45.0	59.8	-8.5	-14.6	-19.7
**TZEEIOR 122 x TZEEIOR 10**	1752	1768	3534	50.4	50.0	-9.6	-11.8	-19.2
**TZEEIOR 122 x TZEEIOR 205**	2378	2097	6244	61.9	66.4	-7.1	-1.3	-7.0
**TZEEIOR 112 x TZEEIOR 43**	2238	2103	5665	60.5	62.9	-6.6	-3.9	-8.8
**TZEEIOR 189 x TZEEIOR 202**	4196	3110	5598	25.0	44.5	5.1	4.1	7.3
**TZEEIOR 195 x TZEEIOR 68**	4056	2387	5353	24.2	55.4	7.7	2.9	9.7
**TZEEIOR 195 x TZEEIOR 149**	4344	2666	5961	27.1	55.3	10.2	4.6	14.0
**TZEEIOR 195 x TZEEIOR 150**	4731	2912	6483	27.0	55.1	9.4	6.8	13.5
**TZEEIOR 82 x TZEEIOR 68**	2293	2686	6933	66.9	61.3	-9.3	3.5	-3.5
**TZEEIOR 82 x TZEEIOR 112**	1629	2745	6471	74.8	57.6	-13.1	2.5	-6.7
**TZEEIOR 30 x TZEEIOR 149**	3072	2843	6880	55.3	58.7	1.4	6.1	9.7
**TZEEIOR 30 x TZEEIOR 150**	4526	2369	5999	24.5	60.5	5.9	1.8	8.1
**TZEEIOR 5 x TZEEIOR 195**	4001	2027	5351	25.2	62.1	6.8	-3.9	6.7
**TZEEIOR 245 x TZEEIOR 195**	3852	3527	5999	35.8	41.2	3.9	7.3	8.5
**TZEEIOR 245 x TZEEIOR 30**	3988	2797	5950	33.0	53.0	4.3	0.7	5.6
**TZEEIOR 253 x TZEEIOR 163**	4720	2238	4824	2.1	53.6	9.7	-2.1	5.0
**TZEEIOR 253 x TZEEIOR 189**	4576	2594	6538	30.0	60.3	5.6	4.9	6.9
**TZEEIOR 132 x TZEEIOR 82**	2117	1835	5301	60.1	65.4	-9.6	-7.0	-12.8
**TZEEIOR 197 x TZEEIOR 163**	4099	2967	5972	31.4	50.3	4.6	4.6	7.0
**TZEEIOR 197 x TZEEIOR 30**	4529	2175	5837	22.4	62.7	6.0	2.9	8.7
**TZEEIOR 212 x TZEEIOR 197**	4216	3215	6281	32.9	48.8	5.7	8.9	11.0
**TZEEIOR 213 x TZEEIOR 197**	4956	2752	5942	16.6	53.7	7.3	4.5	7.1
**TZEEIOR 215 x TZEEIOR 197**	4386	2987	5733	23.5	47.9	4.6	4.3	6.1
**TZEEIOR 217 x TZEEIOR 253**	4349	2994	5526	21.3	45.8	5.4	3.6	4.3
**TZEEIOR 217 x TZEEIOR 197**	5162	2865	5847	11.7	51.0	10.2	5.3	12.1
**TZEEIOR 221 x TZEEIOR 5**	4512	2481	5424	16.8	54.3	11.0	-0.9	9.0
**TZEEIOR 221 x TZEEIOR 197**	4453	2720	5797	23.2	53.1	8.7	3.8	10.8
**TZdEEI 1 x TZdEEI 12 (Check 1)**	3098	1893	5139	39.7	63.2	0.4	-5.2	-2.9
**TZdEEI 7 x TZdEEI 12 (Check 2)**	3185	2340	3192	0.2	26.7	-0.6	1.8	-4.8
**TZdEEI 7 x TZEEI 58 (Check 3)**	3541	2505	5359	33.9	53.3	0.4	2.0	0.7
**TZdEEI 9 x TZEEI 79 (Check 4)**	3802	2499	6807	44.1	63.3	4.3	5.1	8.4
**TZdEEI 12 x TZdEEI 13 (Check 5)**	1813	2408	4674	61.2	48.5	-9.3	1.9	-9.6
**TZdEEI 12 x TZEEI 58 (Check 6)**	3948	2499	5569	29.1	55.1	2.3	-1.7	-0.6
**Mean**	3053	2415	5481	35.5	55.2			
**LSD**	1041	611	849					

STRBI, LNBI and MI means base index for *Striga*, base index for Low-N and Multiple trait base index, respectively.

### Heterotic grouping of inbred lines and identification of testers

**Fig 1:**
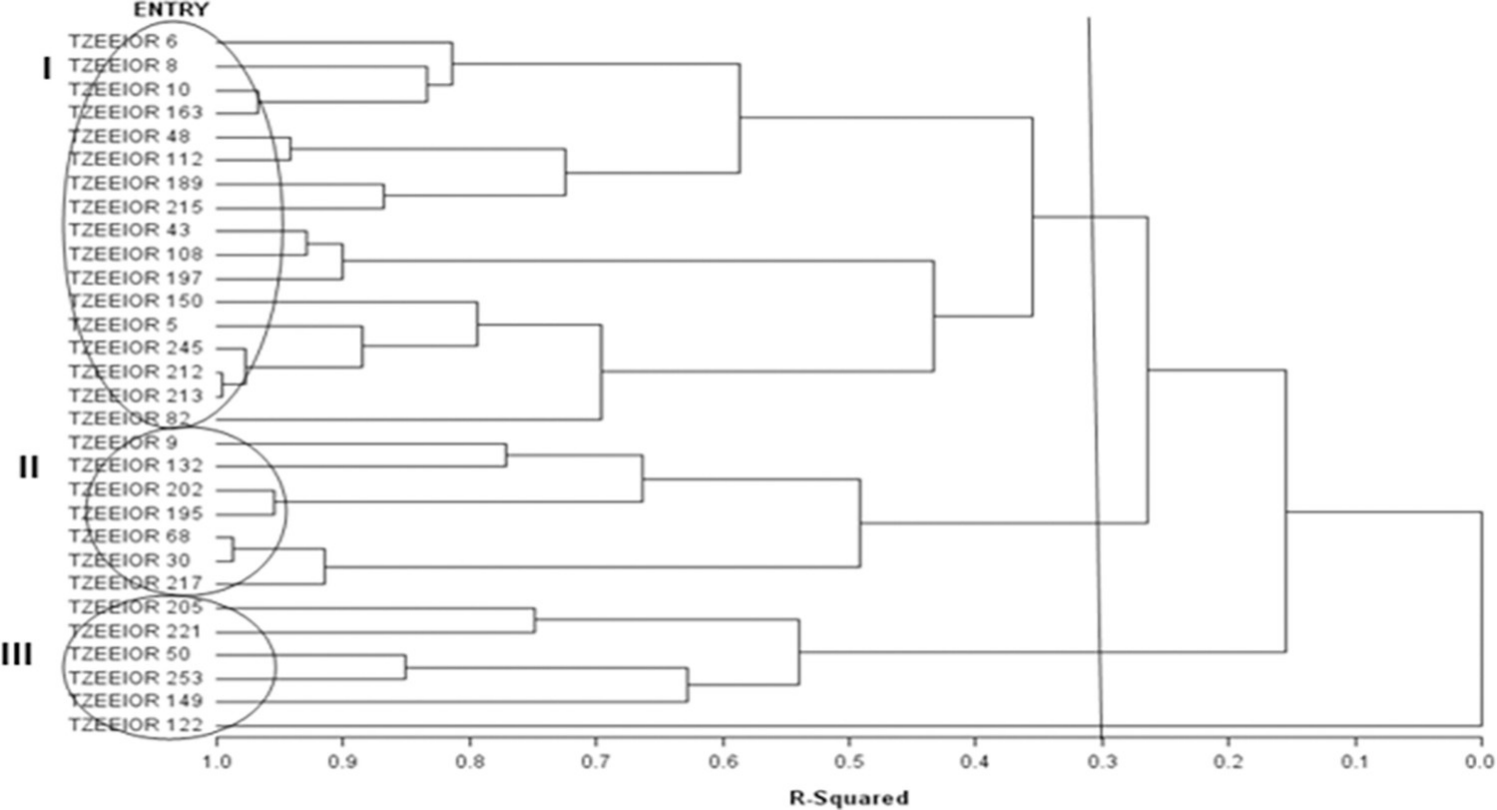
Dendogram of 30 extra-early maturing PVA inbred lines constructed based on the GCA effects of multiple traits (HGCAMT) method across *Striga*, low-N and optimal environments in Nigeria, 2018–2019.

Using the HGCAMT method [12], the 30 inbred lines were categorized into three heterotic groups across the research environments at R-squared value of 30% (Fig 1). Of the 30 PVA inbreds, 17 were clustered in group I. Seven inbred lines (TZEEIOR 9, TZEEIOR 132, TZEEIOR 202, TZEEIOR 195, TZEEIOR 68, TZEEIOR 30 and TZEEIOR 217) were placed in group II while heterotic group III had six inbred lines (TZEEIOR 205, TZEEIOR 221, TZEEIOR 50, TZEEIOR 253, TZEEIOR 149 and TZEEIOR 122). According to Pswarayi and Vivek [41], to qualify as a tester, an inbred must (i) have a high and significant positive GCA effects for grain yield, (ii) belong to a heterotic group, and (iii) possess a high *per se* grain yield. Based on these criteria, TZEEIOR 195 and TZEEIOR 221 were identified as inbred testers for groups II and III, respectively.

Additionally, according to Pswarayi and Vivek [41], a single- cross tester must satisfy the following three criteria (i) parental inbred lines involved in the development of the hybrids must have positive and significant GCA effects for grain yield, (ii) parental lines of the hybrid must belong to the same heterotic group, and (iii) the single-cross hybrid must have a reasonably high grain yield. Therefore, hybrids TZEEIOR 197 x TZEEIOR 82, TZEEIOR 132 x TZEEIOR 195 and TZEEIOR 205 x TZEEIOR 221 from heterotic groups I, II and III, respectively were identified as single-cross testers. These hybrids were selected because the inbred parents that constituted each of them were grouped into the same heterotic group, displayed positive GCA effects for grain yield, and the hybrids were high yielding across the test environments. In addition to high grain yield, the PVA levels of the identified testers were 6.29 μg/g for TZEEIOR 197 x TZEEIOR 82 and 4.62μg/g for TZEEIOR 132 x TZEEIOR 195 while TZEEIOR 205 x TZEEIOR 221 had provitamin A content of 8.30 μg/g (Table 9).

**Table 9:** The PVA contents of selected extra-early single-cross hybrids.

S/N	Materials	α-Carotene (ug/g)	β-Carotene (ug/g)	β- cryptoxanthin (ug/g)	Provitamin A (ug/g)
**1**	**TZEEIOR 163 × TZEEIOR 202**	0.78	4.56	2.63	6.26
**2**	**TZEEIOR 189 × TZEEIOR 202**	1.17	5.63	3.61	8.03
**3**	**TZEEIOR 202 × TZEEIOR 108**	0.99	5.86	0.48	6.60
**4**	**TZEEIOR 202 × TZEEIOR 122**	0.98	4.50	3.93	6.95
**5**	**TZEEIOR 202 × TZEEIOR 48**	1.31	5.77	3.17	8.01
**6**	**TZEEIOR 82 × TZEEIOR 202**	0.91	4.86	4.34	7.49
**7**	**TZEEIOR 108 × TZEEIOR 205**	1.21	4.85	4.80	7.86
**8**	**TZEEIOR 205 × TZEEIOR 212**	1.29	4.99	4.58	7.92
**9**	**TZEEIOR 205 × TZEEIOR 215**	0.85	5.56	3.09	7.54
**10**	**TZEEIOR 205 × TZEEIOR 217**	1.30	5.26	4.41	8.11
**11**	**TZEEIOR 205 × TZEEIOR 221**	1.49	4.92	5.27	8.30
**12**	**TZEEIOR 43 × TZEEIOR 205**	1.21	4.87	4.93	7.94
**13**	**TZEEIOR 48 × TZEEIOR 205**	1.27	5.72	4.39	8.56
**14**	**TZEEIOR 197 × TZEEIOR 82**	0.70	4.24	3.41	6.29
**15**	**TZEEIOR 212 × TZEEIOR 197**	0.85	3.99	3.70	6.27
**16**	**TZEEIOR 221 × TZEEIOR 197**	1.03	4.12	4.16	6.72
**17**	**TZEEIOR 221 × TZEEIOR 5**	0.99	3.68	3.87	6.11
**18**	**TZEEIOR 217 × TZEEIOR 197**	0.77	4.08	3.17	6.05
**19**	**TZEEIOR 9 × TZEEIOR 212**	0.80	3.58	3.95	5.95
**20**	**TZEEIOR 197 × TZEEIOR 30**	0.80	4.17	2.65	5.90
**21**	**TZEEIOR 195 × TZEEIOR 149**	0.82	3.90	3.08	5.85
**22**	**TZEEIOR 112 × TZEEIOR 48**	1.02	3.56	3.52	5.83
**23**	**TZEEIOR 30 × TZEEIOR 149**	0.83	3.78	3.12	5.76
**24**	**TZEEIOR 30 × TZEEIOR 150**	0.77	3.48	3.26	5.49
**25**	**TZEEIOR 50 × TZEEIOR 6**	0.70	3.41	2.87	5.19
**26**	**TZEEIOR 112 × TZEEIOR 50**	0.85	3.12	2.88	4.99
**27**	**TZEEIOR 245 × TZEEIOR 195**	0.64	3.09	2.70	4.76
**28**	**TZEEIOR 132 × TZEEIOR 195**	0.75	2.89	2.72	4.62
**29**	**TZEEIOR 163 × TZEEIOR 149**	0.50	3.34	1.83	4.50
**30**	**TZEEIOR 195 × TZEEIOR 68**	0.49	2.89	2.44	4.35
**31**	**TZEEIOR 195 × TZEEIOR 150**	0.61	2.82	2.27	4.26
**32**	**TZEEIOR 8 × TZEEIOR 217**	0.92	2.35	2.87	4.25
**33**	TZdEEI 7 × TZEEI 58*	0.23	1.44	1.17	2.14
**34**	TZdEEI 9 × TZEEI 79*	0.40	2.23	1.76	3.32
**35**	TZdEEI 12 × TZEEI 58*	0.33	1.33	1.88	2.43
	**Mean**	0.87	3.97	3.23	6.12
	**SE±**	0.05	0.20	0.18	0.28

* Normal yellow endosperm check.

### Inter-trait relationship under *striga-*infested, low-N, and optimal research environments

**Fig 2:**
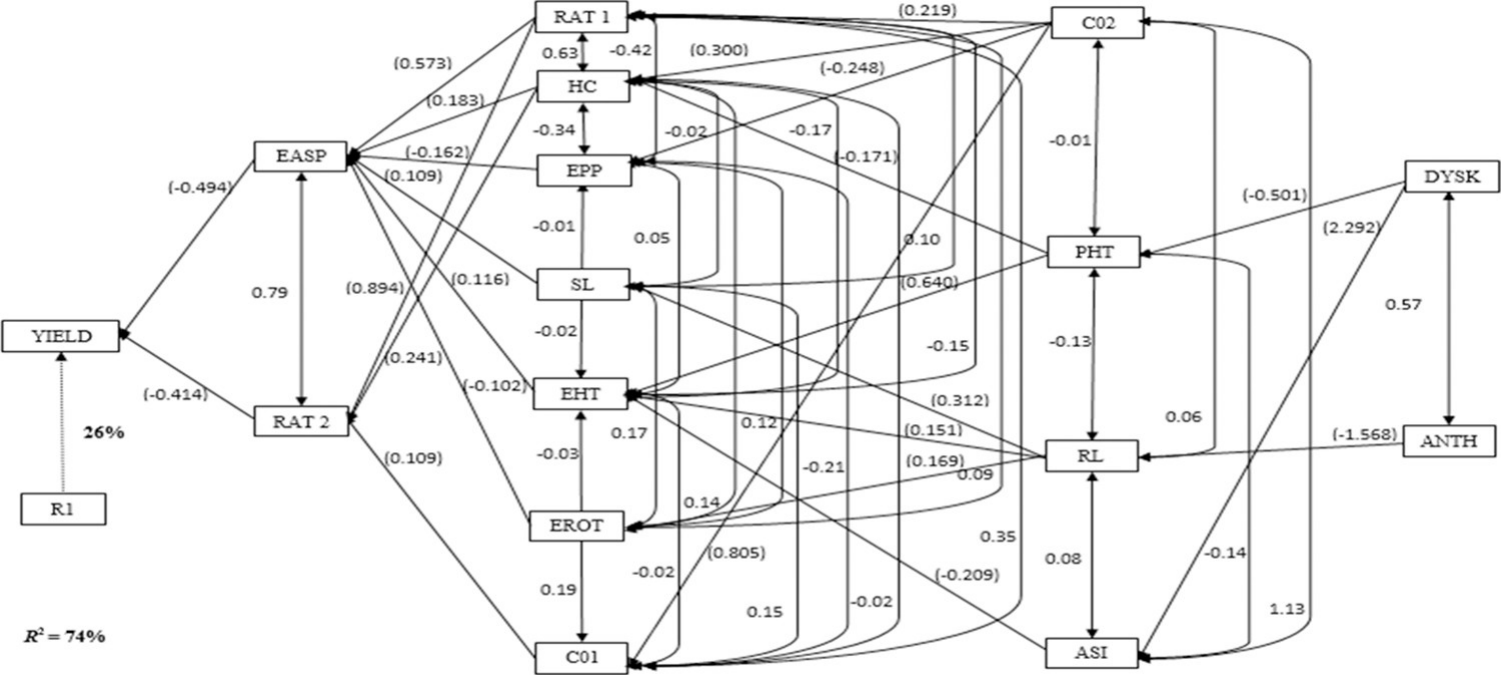
Path analysis model diagram showing causal relationships of measured traits of early maturing provitamin A maize hybrids evaluated under *Striga* infested conditions in Mokwa and Abuja. R^2^ = co-efficient of determination; R1 = residual effects; YIELD = grain yield; EASP = ear aspect; EPP = ears per plant; ANTH = days to 50% anthesis; SL = stalk lodging; ASI = anthesis–silking interval; PASP = plant aspect; DYSK = days to 50% silking; RAT1 and RAT2 = *Striga* damage rating at 8 and 10 WAP, respectively; C01 and C02 = number of emerged *Striga* plants at 8 and 10 WAP, respectively; PHT = plant height; EHT = ear height; HC = husk cover; RL = root lodging; EROT = ear rot.

Under *Striga-*infested environments, the stepwise multiple regression analysis identified EASP and RAT2 as first-order traits with significant direct contributions to grain yield (Fig 2). The two traits accounted for 74% of the total variation in grain yield of the extra-early maturing provitamin A maize hybrids. The EASP and RAT2 had negative direct contribution of -0.494 and -0.414 to grain yield, respectively. Seven traits (RAT1, HC, EPP, SL, EHT, EROT and C01) were categorized as traits with indirect contributions to grain yield through the first-order traits. Apparently, all the second-order traits except C01 had indirect contributions to grain yield through EASP. The RAT1 had positive indirect effects of 0.573 and 0.894 through EASP and RAT2, respectively while EPP (-0.162) had the lowest negative indirect contribution through EASP. Four traits revealed indirect contributions to grain yield through one or more second-order traits. Of the four third-order traits, C02 had the highest positive contribution (0.805) to grain yield through C01 followed by PHT (0.640) through EHT. The DYSK and ANTH were identified as fourth-order traits under *Striga* infested environments. The DYSK (2.292) and ANTH (-1.568) had indirect contributions through ASI while DYSK (0.501) had negative indirect contribution to grain yield through PHT.

**Fig 3:**
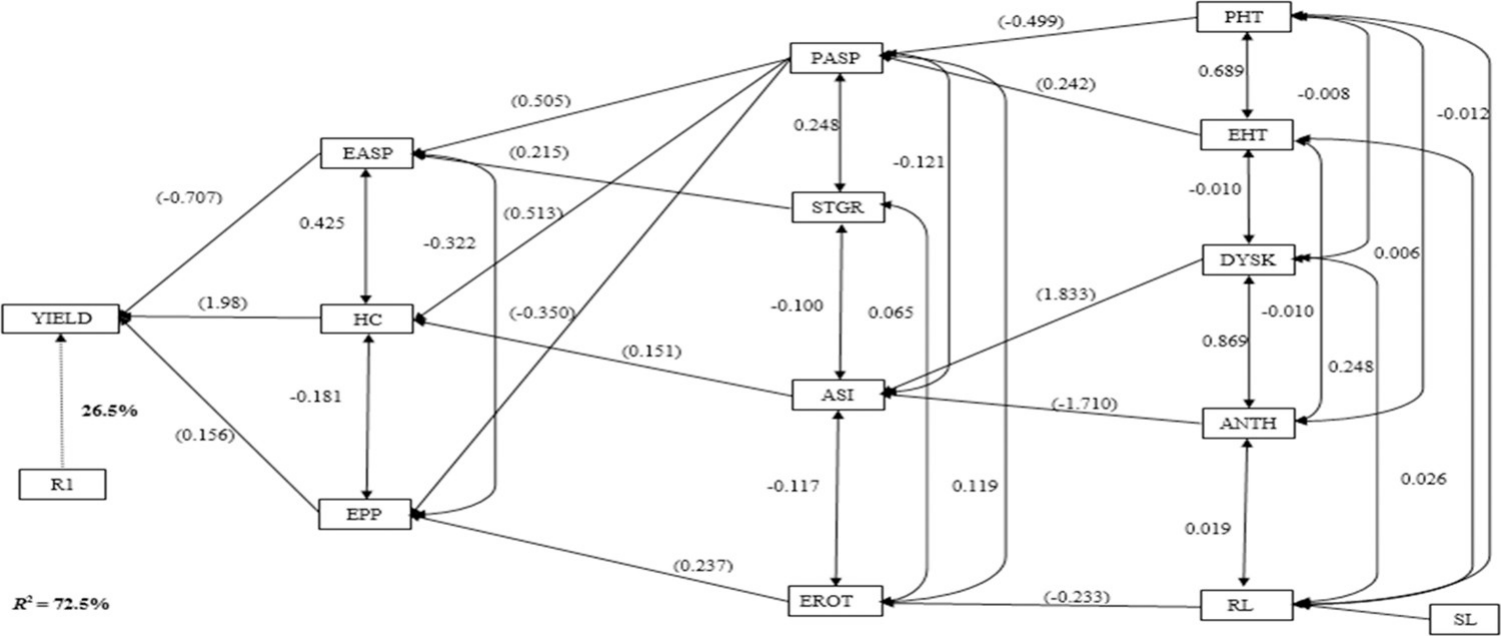
Path analysis model diagram showing causal relationships of measured traits of extra-early maturing provitamin A maize hybrids evaluated under low-N environments in Ile-Ife and Mokwa. R^2^ = co-efficient of determination; R1 = residual effects; YIELD = grain yield; EASP = ear aspect; EPP = ears per plant; ANTH = days to 50% anthesis; SL = stalk lodging; ASI = anthesis–silking interval; PASP = plant aspect; DYSK = days to 50% silking; STGR = stay green characteristics; PHT = plant height; EHT = ear height; HC = husk cover; RL = root lodging; EROT = ear rot.

The EASP, HC and EPP contributed directly to grain yield under low-N (Fig 3). The first-order traits accounted for 72.5% of total variation in grain yield. EASP recorded the highest negative (-0.707) direct contribution to grain yield, while HC (0.198) and EPP (0.156) had significant and positive direct contributions to grain yield. The PASP (0.505 and 0.513) made the highest positive indirect contributions to grain yield through EASP and HC, respectively. In addition, STGR (0.215), ASI (0.151) and EROT (0.237) had positive indirect effects on grain yield through EASP, HC and EPP, respectively. The PASP (-0.350) also had negative indirect contribution to grain yield through EPP. Five traits (PHT, EHT, DYSK, ANTH and RL) constituted the third-order traits. The SL (0.935) was the only trait in the fourth order and had positive contribution to grain yield through RL.

**Fig 4:**
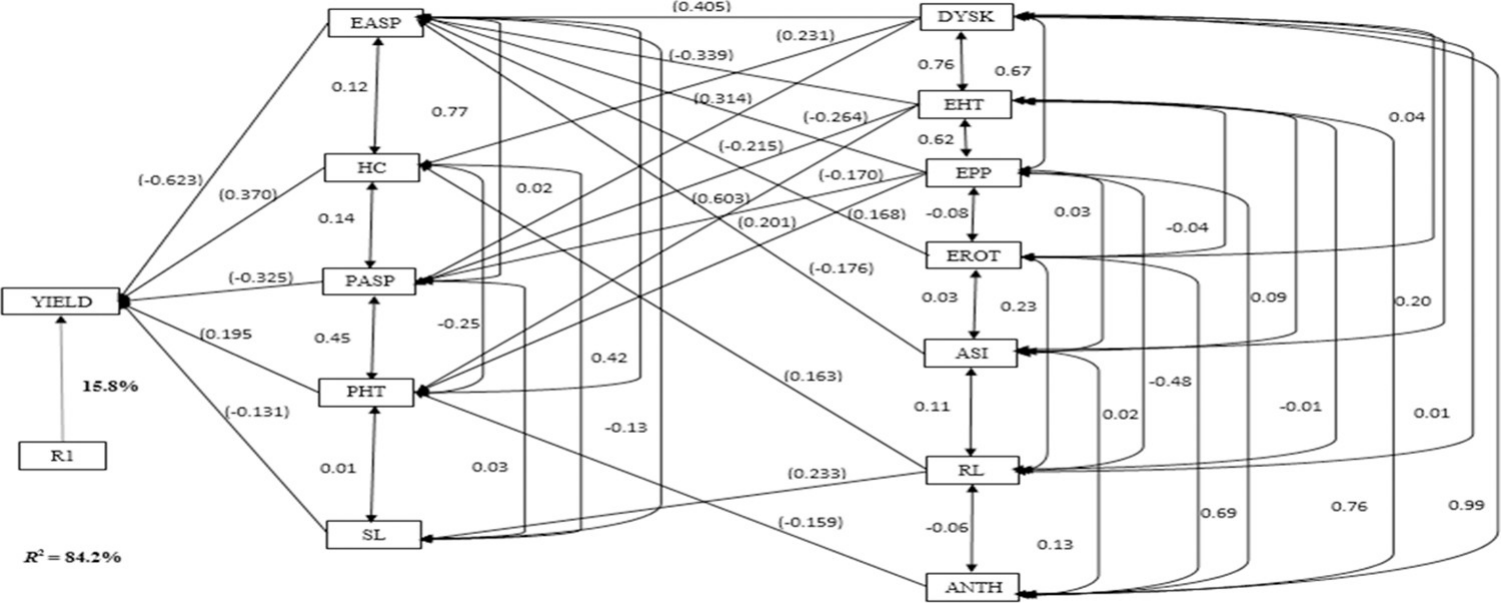
Path analysis model diagram showing causal relationships of measured traits of extra-early maturing provitamin A maize hybrids evaluated under optimal environments in Abuja, Ile-Ife and Mokwa. R^2^ = co-efficient of determination; R1 = residual effects; YIELD = grain yield; EASP = ear aspect; EPP = ears per plant; ANTH = days to 50% anthesis; SL = stalk lodging; ASI = anthesis–silking interval; PASP = plant aspect; DYSK = days to 50% silking; PHT = plant height; EHT = ear height; HC = husk cover; RL = root lodging; EROT = ear rot.

Under optimal environments, the stepwise regression analysis identified EASP, HC, PASP, PHT and SL as first-order traits and explained 84.2% of the variability in grain yield (Fig 4). The HC (0.370) recorded the largest positive path coefficient, whereas EASP had the highest negative path coefficient. The second-order traits identified under optimal environments included DYSK, EHT, EPP, EROT, ASI, ANTH and RL. Each of second-order traits made significant contribution to grain yield through one or more first-order traits. The highest positive indirect effect (0.603) was observed for PHT through PASP, followed by the number of DYSK (0.405), whereas the lowest indirect effect (-0.168) was for EROT through EASP.

### Provitamin A levels of selected hybrids

The provitamin A contents of the selected hybrids ranged from 2.14 for TZdEE7 × TZEEI 58 to 8.56 μgg^-1^ for TZEEIOR 48 × TZEEIOR 205 (Table 9). Of the 35 hybrids subjected to carotenoid analyses, 16 had PVA contents above the mean value of 6.12 μgg^-1^. Crosses involving TZEEIOR 202 had 6 hybrids with PVA levels of between 6.22 μgg^-1^ (TZEEIOR 163 × TZEEIOR 202) and 8.03 μgg^-1^ (TZEEIOR 189 × TZEEIOR 202). There were 7 hybrids involving inbred TZEEIOR 205 with varying PVA level of 7.54 μgg^-1^ for TZEEIOR 205 × TZEEIOR 215 to 8.56 μgg^-1^ TZEEIOR 48 × TZEEIOR 205. However, crosses involving TZEEIOR 197 had 3 hybrids with PVA levels that varied from 6.27 μgg^-1^ for TZEEIOR 212 × TZEEIOR 197 to 6.72 μgg^-1^ TZEEIOR 221 × TZEEIOR 197. The hybrid with the highest PVA content, TZEEIOR 48 × TZEEIOR 205 (8.56 μg/g) out-performed the best normal endosperm check, TZdEEI 9 × TZEEI 79 (3.32 μgg^-1^) by 61%.

### Stability of extra-early maturing PVA maize hybrids across *striga*, low-N and optimal conditions

**Fig 5:**
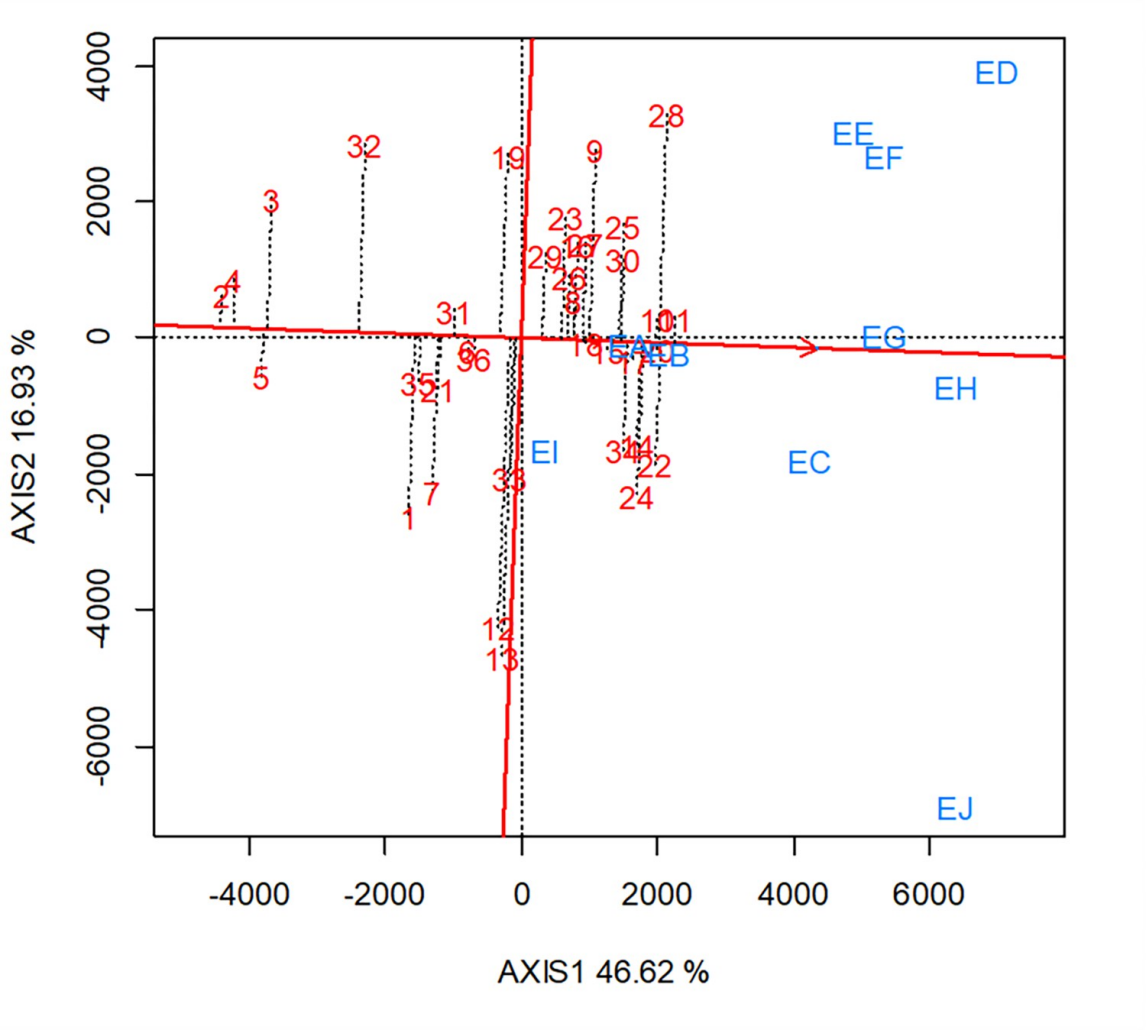
An entry/tester genotype main effect plus genotype x environment biplot showing mean versus stability of grain yield for 30 (best 20 and worst 10 based on the combined *Striga* and low-N base index) selected extra-early maturing provitamin A maize hybrids plus six checks evaluated across *Striga*, low-N and optimal environments from 2018 to 2019 in Nigeria.

The mean performance vs. stability of the GGE biplot view was employed to identify the highest yielding and stable hybrids across the 10 research environments. The average tester coordinate of the biplot separated the genotypes with above mean to the right-hand side of the biplot while those with below grand mean were placed to the left-hand side of the biplot (Fig 5).

List of selected hybrids for stability analysis and the description of the test environments used for the biplot analysis are presented in Table 10A and 10B, respectively. The positions of the hybrids in the biplot were used to determine their yield performance while hybrids’ projections onto the horizontal axis measured the stability. The high-yielding hybrids were positioned far from the vertical axis towards the right-hand side while stable ones had short projections onto the horizontal axis. Therefore, TZEEIOR 195 × TZEEIOR 150 (entry 11) was identified as the top-yielding hybrid followed by TZEEIOR 195 × TZEEIOR 149 (entry 10), TZEEIOR 217 × TZEEIOR 197 (entry 28), TZEEIOR 197 × TZEEIOR 163 (entry 22) and TZEEIOR 253 × TZEEIOR 189 (entry 20) whereas, TZEEIOR 122 × TZEEIOR 6 (entry 2) was the lowest-yielding hybrid. Hybrids TZEEIOR 195 × TZEEIOR 150 (entry 11), TZEEIOR 253 × TZEEIOR 189 (entry 20), TZEEIOR 245 × TZEEIOR 195 (entry 17), TZEEIOR 30 × TZEEIOR 150 (entry 15) and TZEEIOR 245 × TZEEIOR 30 (entry 18) had short projections. The highest-yielding hybrid (entry 11) out-yielded the best check, TZdEEI 9 × TZEEI 79 (entry 34) by 5.47% across the test environments.

**Table 10:** (a): List of selected hybrids for stability analysis; (b) The description of the test environments used for the biplot analysis.

(a)	
ENTRIES	Hybrids
1	TZEEIOR 48 x TZEEIOR 9
2	TZEEIOR 122 x TZEEIOR 6
3	TZEEIOR 122 x TZEEIOR 8
4	TZEEIOR 122 x TZEEIOR 9
5	TZEEIOR 122 x TZEEIOR 10
6	TZEEIOR 122 x TZEEIOR 205
7	TZEEIOR 112 x TZEEIOR 43
8	TZEEIOR 189 x TZEEIOR 202
9	TZEEIOR 195 x TZEEIOR 68
10	TZEEIOR 195 x TZEEIOR 149
11	TZEEIOR 195 x TZEEIOR 150
12	TZEEIOR 82 x TZEEIOR 68
13	TZEEIOR 82 x TZEEIOR 112
14	TZEEIOR 30 x TZEEIOR 149
15	TZEEIOR 30 x TZEEIOR 150
16	TZEEIOR 5 x TZEEIOR 195
17	TZEEIOR 245 x TZEEIOR 195
18	TZEEIOR 245 x TZEEIOR 30
19	TZEEIOR 253 x TZEEIOR 163
20	TZEEIOR 253 x TZEEIOR 189
21	TZEEIOR 132 x TZEEIOR 82
22	TZEEIOR 197 x TZEEIOR 163
23	TZEEIOR 197 x TZEEIOR 30
24	TZEEIOR 212 x TZEEIOR 197
25	TZEEIOR 213 x TZEEIOR 197
26	TZEEIOR 215 x TZEEIOR 197
27	TZEEIOR 217 x TZEEIOR 253
28	TZEEIOR 217 x TZEEIOR 197
29	TZEEIOR 221 x TZEEIOR 5
30	TZEEIOR 221 x TZEEIOR 197
31	TZdEEI 1 x TZdEEI 12 Check 1
32	TZdEEI 7 x TZdEEI 12 Check 2
33	TZdEEI 7 x TZEEI 58 Check 3
34	TZdEEI 9 x TZEEI 79 Check 4
35	TZdEEI 12 x TZdEEI 13 Check 5
36	TZdEEI 12 x TZEEI 58 Check 6
(b)	
Environments	Description
EA	Ile-Ife Low-N, 2018
EB	Mokwa Low-N, 2018
EC	Mokwa Low-N, 2019
ED	Abuja *Striga*, 2018
EE	Mokwa *Striga*, 2018
EF	Mokwa *Striga*, 2019
EG	Ile-Ife optimal, 2018
EH	Abuja optimal, 2018
EI	Mokwa optimal, 2018
EJ	Mokwa optimal, 2019

## Discussion

The differences in the performance of the hybrids from one research condition to the other i.e *Striga*, low-N and optimal environments suggested that the hybrids responded differently to the contrasting environmental conditions. This indicated the uniqueness of the contrasting test environments which revealed the genetic properties of the hybrids [7,42,43]. Oyekale et al. [44] also reported significant differences among extra-early PVA-quality protein hybrid maize under *Striga* and low-N stresses in Nigeria.

The significant GCA-male and/ or GCA-female variances observed for grain yield and other measured agronomic traits under *Striga*, low-N and optimal environments revealed the preponderance of additive genetic variances in the inheritance of these traits. The observed significant SCA variances for RAT1, C01, C02 and EASP under *Striga*, grain yield under low-N and optimal conditions implied that non-additive genetic actions were also important for these traits. These findings suggested that recurrent selection and hybridization would be invaluable for the genetic enhancement of *Striga* tolerance/ resistance, low-N tolerance and *per se* performance of progenies in a population derived from the extra-early PVA materials evaluated. This corroborated the earlier reports by Laban et al. [45], that both additive and non-additive genetic actions moderated the inheritance of grain yield and other agronomic traits of the early maturing provitamin A maize inbreds evaluated across *Striga*-infested and optimal growing environments in Nigeria. Similarly, Oyekale *et al*. [44] reported the importance of additive and non-additive gene action in the inheritance of grain yield and some other traits in the extra-early PVA-QPM hybrids under *Striga*, low-N and optimal environments.

The presence of genotype × environment interactions for grain yield, *Striga* damage ratings and emerged *Striga* plants under *Striga* infestation indicated that the hybrids reacted differently to *Striga* infestation at various locations, thus suggesting differential reaction of the hybrids to *Strig*a. Similar findings have been reported by several authors [11,26,46–48]. The significant effects of env × hybrid observed for grain yield and most measured traits under the contrasting environment suggested that there were differential responses among the hybrids to the environments [32,49]. It is therefore, imperative for researchers to carry out evaluations in several environments [30,50] and assess the yield performance and stability of genotypes using the available statistical tools [50,51] to select desirable genotypes. However, the non-significant ASI and STGR for GCA-male, ASI for GCA-female (under low-N), EPP for GCA-male and ASI for GCA-female under optimal environments indicated that the inbreds reacted consistently as parents in hybrid combinations for ASI, STGR and EPP under low-N and optimal conditions, respectively. Also, the non-significance of ASI for hybrid, GCA-male, GCA-female and SCA under low-N inferred that ASI did not have significant effects among the PVA hybrids under low-N environments. This implied that the inclusion of ASI in the base index under low-N environments was not important. These findings contradicted the conclusions of earlier authors [52,53] who identified ASI as an important trait for indirect selection under low-N environments when grain yield improvement was the objective. It is however, consistent with the findings of Badu-Apraku and Fakorede [54] and Obeng-Bio et al. [55] who reported that ASI is inconsequential for indirect selection for grain yield in maize under low-N environments. The disparity in these findings and earlier studies by Bolaños and Edmeades [52] and Bänziger et al. [53] could be attributed to differences in the maturity group of the genetic materials used in the studies. The larger proportion of total variation due to environment relative to genotype and genotype × environment interaction observed under *Striga*, low-N and optimal environments was an indication that, like other maize cultivars, multi-environment evaluation over years is necessary for PVA maize hybrids prior to advancement for commercialization [48,56–58].

The significant GCA-male × Env, GCA-female × Env and SCA × Env reported for grain yield and most other measured traits under *Striga*, low-N and optimal environments revealed that there were disparities in the additive variances from one environment to the other and that both additive and non-additive effects were important for grain yield. Badu-Apraku et al. [12], Obeng-Bio et al. [55] and Makumbi et al. [58] also reported significant GCA × Env interaction variances for grain yield and some other traits of maize under contrasting research environments.

The significant GCA (GCA-male and GCA-female_)_ and SCA for grain yield and most other measured traits under low-N conditions, was an indication that additive and non-additive variances were both important in the inheritance of grain yield and some other agronomic traits under low-N. Nasser et al. [59], Olayiwola et al. [60] and Osuman et al. [61] also reported significant GCA and SCA variances for maize grain yield and other agronomic traits under various stress conditions. The non-significant SCA variances for grain yield, ASI, RAT2 and EPP under *Striga* environments; ASI, EPP and STGR under low-N; ASI, EPP and EASP under optimal environments indicated that non-additive gene action was not important in the inheritance of those traits in the evaluated inbred lines. Badu-Apraku et al. [12] and Ifie et al. [57] reported non-significant SCA variances for the STGR in early maturing maize under low-N conditions.

The preponderance of GCA sum of squares over their corresponding SCA sums of squares for grain yield and some other traits under *Striga* and optimal conditions confirmed the importance of additive gene action over non-additive gene action in the inheritance of those traits in the set of inbred lines. This finding agrees with the reports of Badu-Apraku et al. [31]; Badu-Apraku and Oyekunle, [42] who reported the importance of additive gene action for *Striga* resistance and drought tolerance in early and extra-early maize inbred lines, respectively. Ifie et al. [57] also reported additive genetic variances for *Striga* resistance and low-N tolerance for early white maize inbred lines. Contrarily, non-additive gene action was more important than the additive gene action for grain yield and most other measured traits under low-N conditions. This corroborated the findings of Makumbi et al. [58] and Meseka et al. [62] that non-additive gene action was more important in the inheritance of grain yield under low-N conditions. It however contradicted the reports of other scholars [57,63–65] that additive gene action was more important in the inheritance of grain yield under low-N conditions.

The superiority of NCD II lies in the ability to accommodate more parental lines and allow for the estimation of maternal and paternal effects as against the diallel method [55,66]. When the value of GCA-female is greater than the values of GCA-male the implication is that the cytoplasm had a genetic factor, which might have influenced the expression of such traits in addition to nuclear genes [44]. The results of the present study showed that the ratio of GCA-female to that of GCA-male was greater than 1 for grain yield, and most other measured traits under *Striga* infestation, low-N and optimal environments. This indicated that maternal effects contributed more to the expression of those traits in each of the research conditions. Under the *Striga* environments, paternal effects controlled the inheritance of number of emerged *Striga* plants at 8 WAP because the ratio of GCA-female sum of squares to GCA-male sum of squares was less than 1. Similarly, paternal effects controlled the inheritance of anthesis-silking interval under low-N stress and optimal conditions. The larger GCA-female sum of squares compared to GCA-male for *Striga* damage, number of emerged *Striga* plants, plant aspect, number of ears per plant (prolificacy), ear aspect and the stay green characteristic suggested that a cautious selection of female parents is required to exploit favourable cytoplasmic effects for those traits. The results of this study agreed with those of Oyekunle and Badu-Apraku [67] who reported maternal effects for grain yield in early-maturing maize under optimal environments. Oyekale et al. [44] also reported maternal effects for grain yield of extra-early maturing PVA QPM under *Striga*, low-N and high-N conditions. Contrarily, Ifie et al. [57] reported similar contributions of GCA-female and GCA-male to grain yield and most other measured traits of early maturing maize genotypes under *Striga*, low-N and optimal environments. The differences in the results of Ifie et al. [57] and the present study could be attributed to differences in the genetic materials under evaluation, the severity of the imposed stresses as well as the differences in the environments.

The success of any crop breeding programme greatly depends on the ability of the parental lines to transfer favourable alleles to their progenies [17]. Thus, the available genetic materials within the crop improvement programme should pass through serious selection processes to determine their intrinsic values [68]. Combining ability studies involve determining the average breeding value of the germplasm used as well as the genetic value due to the interaction between these specific genes in cross combinations (specific combining ability) [68,69]. The specific combining ability is due to non-additive (dominance) gene action while the general combining ability is due to the additive gene action [69]. A parental line with significant and positive GCA effects for grain yield under stress (e.g *Striga* and low-N) has high prospect of donating favourable alleles for grain yield to the progenies in a recurrent selection programme [70]. Such parent(s) are useful in population improvement and for development of *Striga* and or low-N tolerant varieties [70]. The significant positive GCA-male effects recorded for grain yield for the parental lines TZEEIOR 205, TZEEIOR 150, TZEEIOR 82 and TZEEIOR 221 and GCA-female effects displayed by TZEEIOR 9, TZEEIOR 108, TZEEIOR 189, TZEEIOR 195 and TZEEIOR 253 under *Striga* environments indicated that these inbred lines would donate complimentary alleles for grain yield under *Striga* infestation, when used as males and females, respectively. Inbred lines TZEEIOR 205, TZEEIOR 150, TZEEIOR 202, TZEEIOR 195, TZEEIOR 82 and TZEEIOR 221 with significant and negative GCA-male effects for *Striga* damage and TZEEIOR 108, TZEEIOR 149, TZEEIOR 150, TZEEIOR 253 and TZEEIOR 221 with significant and negative GCA-female effects for *Striga* damage will contribute significantly to reduction of *Striga* damage when used as male and female parents, respectively in hybrid production. Contrarily, TZEEIOR 6, TZEEIOR 253, TZEEIOR 8, TZEEIOR 149, TZEEIOR 150, TZEEIOR195 and TZEEIOR 221 that exhibited significant and negative GCA-male and GCA-female effects for number of emerged *Striga* plants at 10 WAP, respectively will be desirable in the development of *Striga* resistant hybrids as they will transfer *Striga* resistance to their progenies when used as male or female parents. Inbreds TZEEIOR 205 and TZEEIOR 221 with positive and significant GCA-male effects for grain yield and TZEEIOR 48 with significant positive GCA-female effects for grain yield under low-N will contribute favourable alleles for grain yield under low-N conditions. The negative and significant GCA-female effects for STGR observed for TZEEIOR 6 suggested that this inbred line possessed alleles that delayed leaf senescence. Therefore, TZEEIOR 6 should be selected as female parent in hybrid programmes to transfer the alleles to the offspring.

The recorded average grain yield reduction under *Striga* infestation fell within the range of 30–90% reported by different scholars [31,69,71,72]. These results confirmed that the *Striga* infestation level was severe enough to efficiently discriminate among the hybrids. Therefore, any genotype identified as *Striga* resistant would support considerably less *Striga* plants and yield higher than the susceptible ones. In contrast, a genotype that was classified as tolerant to *Striga* was expected to support as many *Striga* plants as the susceptible genotypes but would produce higher grain and stover than the susceptible genotypes while showing fewer damage symptoms [56]. Using the *Striga* selection base index, hybrid TZEEIOR 221 × TZEEIOR 5 was identified as the highest yielding and resistant to *Striga*. Other top-performing hybrids included TZEEIOR 217 × TZEEIOR 197, TZEEIOR 195 × TZEEIOR 149 and TZEEIOR 253 × TZEEIOR 163. These hybrids were high-yielding and supported fewer *Striga* plants under *Striga* infestation. It is striking to note that about 6.1% *Striga* emergence reduction was observed on TZEEIOR 197 × TZEEIOR 30 between 8 and 10 WAP. This suggested that *Striga* resistance alleles could be present in one or both parents that constituted this hybrid. It is important to note that inbred lines that are *Striga* resistant could be extracted from this hybrid using bi-parental crosses followed by a pedigree selection scheme. Additionally, *Striga* resistance genes from the inbreds TZEEIOR 30 and TZEEIOR 197 could be introgressed into breeding populations while the hybrid (TZEEIOR 30 × TZEEIOR 197) could be widely tested and deployed to *Striga* endemic areas to reduce *Striga* seed bank and combat malnutrition in SSA.

The average grain yield reduction of 54% reported in the present study under low-N was higher than the 36% reported by Obeng-Bio et al. [55]. However, Wolfe et al. [73] and Bänziger et al. [74] reported 10–50% and 20–50% yield reduction under low-N environments, respectively. This implied that the imposed stress was severe enough to successfully discriminate among the low-N tolerant and susceptible PVA hybrids. The low-N base index [30] that combined grain yield, ears per plant, anthesis-silking interval, plant and ear aspects and the stay-green characteristic was effectively utilized to select outstanding hybrids. The top performing hybrids under low-N conditions were TZEEIOR 212 × TZEEIOR 197, TZEEIOR 245 × TZEEIOR 195, TZEEIOR 195 × TZEEIOR 150, TZEEIOR 30 × TZEEIOR 149 and TZEEIOR 217 × TZEEIOR 197. The top ranking PVA hybrid (TZEEIOR 212 × TZEEIOR 197) out-yielded the best yellow endosperm hybrid check TZdEEI 9 × TZEEI 79 by 22.3%.

The assumption was that the top performing hybrids under *Striga* and/ low-N would also produce reasonable grain yield under optimal environments, this assumption may not hold in many cases. Thus, focus was made in this study to ensure that hybrids with outstanding performance under *Striga* and/ low-N would not carry yield penalties under non-stress conditions. Multiple trait base index was used to rank the hybrids under optimal environments while placing more emphasis on the performance under stress (*Striga* and low-N) conditions. Hybrids TZEEIOR 195 × TZEEIOR 149, TZEEIOR 195 × TZEEIOR 150, TZEEIOR 217 × TZEEIOR 197, TZEEIOR 212 × TZEEIOR 197 and TZEEIOR 221 × TZEEIOR 197 were identified as the top-yielding hybrids under optimal conditions. The yield of the top-performing PVA hybrid, TZEEIOR 195 × TZEEIOR 149 (5961 kg ha^-1^) was statistically similar to the grain yield of the best yellow endosperm check, TZdEEI 9 × TZEEI 79. Although, the best check out-yielded the top-ranking hybrid by 12.43%, it did not possess other desirable traits under *Striga* and low-N as the former. The implication is that the PVA hybrid TZEEIOR 195 × TZEEIOR 149 did not only have outstanding yield under optimal conditions, but also combined *Striga* resistance with low-N tolerance.

Grouping of the newly developed extra-early maturing PVA inbred lines into appropriate heterotic groups is important for exploring hybrid vigour because of inter-group mating of inbreds [75–77]. This would increase the chances of developing novel and superior extra-early maturing PVA hybrids and synthetics with combined *Striga* and low-N tolerance for commercialization in SSA. Even though the extra-early maturing PVA inbred lines used in this study were bred from one source population, they were clustered into three heterotic groups using the heterotic grouping based on GCA of multiple traits (HGCAMT) method proposed by Badu-Apraku et al. [12]. The results revealed that broad genetic diversity existed among the extra-early maturing PVA inbred lines. Laban et al. [44] also classified early maturing PVA inbred lines that were developed from the same source population into different heterotic groups. This confirmed that broad genetic diversity within the extra-early maturing source population existed. The population from which the extra-early maturing PVA maize lines were extracted was developed from a mixture of PVA germplasm of different genetic composition through introgression followed by a cycle of backcrossing, selfing and recombination [26]. The different breeding methodologies employed in developing the source population may have contributed to the wide genetic base. Inter-heterotic mating of these inbred lines might have resulted in a highly vigorous and genetically diverse population for enhanced maize production in SSA. The inbred lines that were classified into the same heterotic group in this study could be recombined to form heterotic populations which could be improved through recurrent selection methods. The identified inbred testers (TZEEIOR 195 and TZEEIOR 221) could be used to classify other extra-early maturing PVA inbred lines into heterotic groups. Single-cross hybrid testers, TZEEIOR 197 × TZEEIOR 82, TZEEIOR 132 × TZEEIOR 195 and TZEEIOR 205 × TZEEIOR 221 would serve as outstanding female parents for development of 3-way and double-cross PVA hybrids.

The yield performance of maize is generally the result of complex interactions among secondary traits and environments. The stepwise multiple regression and sequential path coefficient analyses enhanced the understanding of the influences of different secondary traits on grain yield [38,56]. The two analyses effectively established the relative importance of the yield-related traits involved in the analysis and measured their direct and indirect contributions to the main response variable, the grain yield. Under *Striga* environments, the total variation of 74% explained in the present study suggested that the traits involved in the path coefficient analysis accounted for a substantial variation in grain yield. The direct contributions of EASP and RAT2 to grain yield revealed that these traits were the most important determinants of grain yield in *Striga* endemic environments. This finding further justified the inclusion of EASP and *Striga* damage employed in the *Striga* resistance base index. This means that genotypes selected under *Striga* environments based on these traits would be more productive. The RAT1, HC and C01 had indirect effects on grain yield through RAT2 whereas six of the seven second-order traits (RAT1, HC, EPP, SL, EHT and EROT) contributed to grain yield through EASP, thus emphasizing the significance of *Striga* damage rating and EASP in the genetic enhancement of maize hybrids for *Striga* resistance. While C02, PHT, RL and ASI were identified as third-order traits, C02 had significant contribution to grain yield through four of the seven second-order traits (RAT1, HC, EPP and C01), suggesting that this trait is a very important regulator of grain yield under *Striga* infestation.

Under low-N conditions, EASP, HC and EPP contributed directly to grain yield. This finding highlighted the importance of EASP and EPP as key traits determining grain yield under low-N conditions. Talabi et al. [38] identified EASP and EPP as the first-order traits while Obeng-bio et al. [55] identified EASP only as among the first-order traits contributing to grain yield under low-N environments. Similarly, PASP, STGR and ASI were among the important traits for grain yield selection under low-N conditions, as they were identified as second-order traits. This agreed with the findings of earlier researchers [11,12,38,78] who identified the STGR, EASP and ASI as the most important secondary traits under low-N. The results also corroborated the report of Obeng-bio et al [55], who identified STGR and ASI as second-order traits of early PVA hybrids. The high co-efficient of determination (r^2^) of 72.5% in the present study suggested that the traits used were effective in accounting for a substantial variation in grain yield. The EASP, HC, PASP, PHT and SL revealed direct contributions to grain yield under optimal conditions. This indicated that the PVA hybrids relied on the nice appearance of the ears, stay-green characteristic, closed ear-tips, photosynthetic ability, and plant standability for increased grain yield.

The selected hybrids based on high grain yield across environments possessed PVA contents below the recommended level of 15.00 μg g^−1^ [79]. Nevertheless, hybrids such as TZEEIOR 189 × TZEEIOR 202 (8.03 μg g^−1^), TZEEIOR 205 × TZEEIOR 217 (8.11 μg g^−1^), TZEEIOR 202 × TZEEIOR 221 (8.30 μg g^−1^), TZEEIOR 48 × TZEEIOR 205 (8.56 μg g^−1^) and TZEEIOR 202 × TZEEIOR 48 (8.01 μg g^−1^) had intermediate PVA levels. It is interesting to note that, the levels of PVA contents of the hybrids reported in the present study were lower than the 20.1 and 22.7 μg g^−1^ reported for TZEEIOR 197 × TZEEIOR 205 and TZEEIOR 202 × TZEEIOR 205, respectively by Badu-Apraku et al. [48] but comparable with the 3–8 μg g^−1^ reported for the available orange endosperm maize [55,79]. Therefore, the identified hybrids could be promoted for commercialization to improve nutrition in SSA.

The yield performance of maize hybrids and its stability are invaluable in multi-environment evaluations. Therefore, a major goal of the present study was to identify high-yielding and stable PVA hybrids across *Striga-*infested, low-N and optimal environments for commercialization in SSA. There is the need for adoption of hybrids with wide adaptation by farmers in SSA because of the large variations in low-N levels in tropical soils and consumer preferences. However, location-specific maize hybrids could also be of great importance in some cases. The significant genotype × environment interaction observed for grain yield under *Striga*, low-N and optimal environments indicated that the expression of the traits differed in the contrasting environments. Similar findings were reported in earlier studies conducted in hybrid maize under stress and stress-free environments [80,81]. The GGE biplot identified TZEEIOR 195 × TZEEIOR 149, TZEEIOR 195 × TZEEIOR 150, TZEEIOR 245 × TZEEIOR 195, TZEEIOR 30 × TZEEIOR 150 and TZEEIOR 245 × TZEEIOR 30 as high yielding and stable hybrids across *Striga*, low-N and optimal growing conditions. These hybrids would contribute immensely to food security and nutrition when commercialized in SSA.

## Conclusions

Both additive and non-additive gene action played major roles in the inheritance of grain yield and most measured traits in this study. However, since there was preponderance of additive over non-additive gene action under *Striga* and optimum conditions, recurrent selection could be employed for population improvement followed by the extraction of *Striga* resistant inbred lines with high combining ability effects for hybrid development. Non-additive gene action was more important than the additive in the inheritance of grain yield under low-N environments, justifying the need for hybridization in the program for the development of low-N tolerant maize hybrids. Since maternal effects influenced the inheritance of grain yield and several other important traits, in selection of female parents for hybrid development, advantage should be taken of favourable cytoplasmic effects in selecting parents for hybridization. The consistent direct positive contribution of ear aspect to grain yield across *Striga*, low-N and optimal environments justified its inclusion in the multiple trait base index for selection of *Striga* resistant and low-N tolerant maize genotypes. The inbred testers identified in the present study could be used to classify other PVA inbred lines of IITA into heterotic groups while the single cross testers could be used as female parents in developing high yielding 3-way cross PVA hybrids. Hybrids with moderate PVA contents should be invaluable in combating malnutrition in SSA. In addition, outstanding PVA hybrids identified in this study should be evaluated in on-farm trials and promoted for commercialization to reduce malnutrition and food security challenges in SSA.
